# Efficient Implementation of the Spin-Free Renormalized Internally-Contracted Multireference Coupled Cluster Theory

**DOI:** 10.1021/acs.jpca.5c07588

**Published:** 2026-02-03

**Authors:** Kalman Szenes, Riya Kayal, Kantharuban Sivalingam, Robin Feldmann, Frank Neese, Markus Reiher

**Affiliations:** † ETH Zürich, 31064 Department of Chemistry and Applied Biosciences, Vladimir-Prelog-Weg 2 8093 Zürich, Switzerland; ‡ 28314 Max-Planck-Institut für Kohlenforschung, Kaiser-Wilhelm-Platz 1 45470 Mülheim an der Ruhr, Germany

## Abstract

In this paper, an efficient implementation of the renormalized internally contracted multireference coupled cluster with singles and doubles (RIC-MRCCSD) into the ORCA quantum chemistry program suite is reported. To this end, Evangelista’s Wick&d equation generator was combined with ORCA’s native AGE code generator in order to implement the many-body residuals required for the RIC-MRCCSD method. Substantial efficiency gains are realized by deriving a spin-free formulation instead of the previously reported spin–orbital version developed by some of us. Since AGE produces parallelized code, the resulting implementation can directly be run in parallel with substantial speedups when executed on multiple cores. In terms of runtime, the cost of RIC-MRCCSD is shown to be between single-reference RHF-CCSD and UHF-CCSD, even when active space spaces as large as CAS­(14,14) are considered. This achievement is largely due to the fact that no reduced density matrices or cumulants higher than three-body enter the formalism. The scalability of the method to large systems is furthermore demonstrated by computing the ground-state of a vitamin B_12_ model comprised of an active space of CAS­(12,12) and 809 orbitals. In terms of accuracy, RIC-MRCCSD is carefully compared to second- and approximate fourth-order *n*-electron valence state perturbation theories (NEVPT2, NEVPT4­(SD)), to the multireference zeroth-order coupled-electron pair approximation (CEPA(0)), as well as to the IC-MRCCSD from Köhn. In contrast to RIC-MRCCSD, the IC-MRCCSD equations are entirely derived by AGE using the conventional projection-based approach, which, however, leads to much higher algorithmic complexity than the former as well as the necessity to calculate up to the five-body RDMs. Remaining challenges such as the variation of the results with the flow, a free parameter that enters the RIC-MRCCSD theory, are discussed.

## Graphical abstract

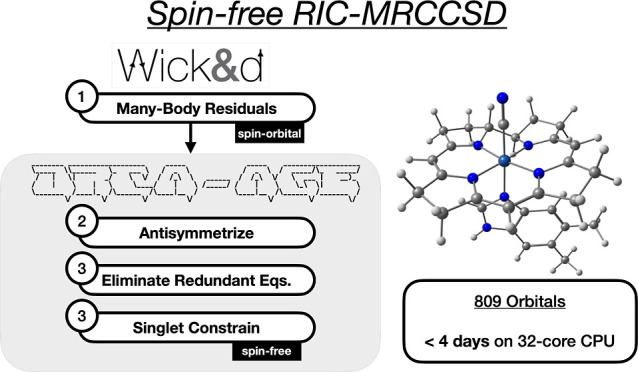

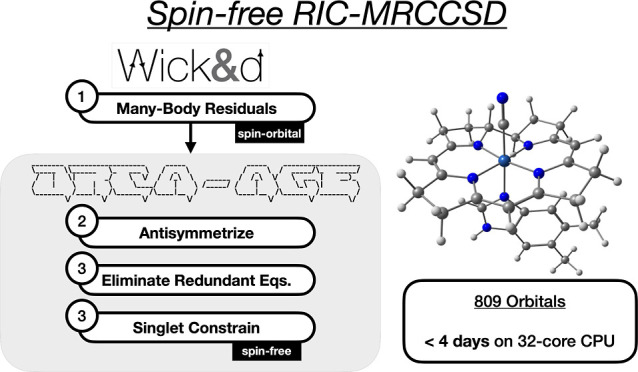

## 1 Introduction

The electronic structure of closed-shell molecules can be accurately described by coupled cluster theory,^1^ which relies on the mean-field Hartree–Fock determinant as a zeroth-order approximation. However, for many chemically relevant systems, such as transition-metal complexes and biradicals, the mean-field solution does not dominate the full wave function and, consequently, methods built upon it suffer in terms of reliability and accuracy. Therefore, such systems are typically described with active orbital space methods, which partition the orbitals into subspaces, of which one is usually treated exactly while the remaining orbitals are neglected.

If the orbitals are chosen carefully, such that strongly correlated orbitals are included in the subspace, this scheme can provide a qualitative description of the full electronic structure. However, to obtain accurate properties, such active space methods need to be complemented by multireference schemes, which attempt to recover the electron correlation from the full set of orbitals, including the neglected orbitals. The workhorse of these methods in chemistry is second-order perturbation theory, for which complete active space (CASPT2)^2^ and *n*-electron valence state perturbation theory (NEVPT2)^3^ are the two prime examples. These methods are currently considered state of the art despite providing only low-order perturbative corrections.

By contrast, a variety of multireference coupled cluster methods exists to address complicated open-shell systems, ranging from an uncontracted ansatz (Jezorski-Monkhorst)^4^ to fully internally contracted ones.^5–7^ These methods are computationally demanding and thus limited to niche applications.^8,9^ Among them, the internally contracted MRCC (IC-MRCC) developed by Köhn and co-workers presents a very general formalism.^5,10–14^ However, the ansatz leads to reduced density matrices of up to fifth order, which severely limits its application to small active spaces. Internally contracted coupled-electron pair approximation (CEPA) approaches are more affordable and similar in quality, although some variants may lack formal properties such as size-consistency or orbital invariance.^15^

Higher order reduced matrices can be avoided by exploiting many-body residuals, as in the partially internally contracted MRCC approach of Datta and Nooijen.^16^ However, this formalism can suffer from convergence issues, as reported by Lechner and co-workers.^17^ Driven similarity renormalization group (DSRG) approaches, developed in the group of Evangelista, mitigate these issues by regularizing diverging amplitudes.^18–20^ In ref 21, some of us introduced the renormalized internally contracted multireference coupled cluster (RIC-MRCC) theory, a novel multireference approach that adapts the DSRG scheme to nonunitary similarity transformations, thereby resembling conventional MRCC methods.^5,22–28^ Its defining characteristics can be summarized as follows:

1. RIC-MRCC relies on the internally contracted ansatz, where a single cluster operator is applied on the entire CAS reference wave function.
2. It relies on many-body residuals^16^ based on the generalized normal-ordering of Mukherjee and Kutzelnigg,^29,30^ which correspond to the matrix elements of the effective Hamiltonian. This yields a simpler set of residual equations compared to the conventional ones obtained from the projected residuals. These residuals are devoid of linear dependencies, which are inherent to the internally contracted ansatz^2,31^ and plague MRCC schemes in particular.^14,32,33^

3. To simplify the working equations, a large number of contractions involving amplitudes with multiple active indices have been neglected. As a consequence, RIC-MRCC only relies on up to three-body reduced density matrices and cumulants, making it amenable to large active space calculations.
4. The update equation for the amplitudes is augmented by a regularization factor, which attempts to remove numerical instabilities. This factor, however, introduces a free parameter which is examined in this study.

In this paper, we present an implementation of the RIC-MRCC method with single and double excitations in the ORCA^34^ quantum chemistry package. By contrast to the initial publication,^21^ which was expressed in a spin–orbital basis, we reformulate all equation in spin-free form. To achieve this, the many-body residual equations obtained from the Wick&d^35^ program have been translated to ORCA’s internal code generator AGE’s^36^ format, which subsequently carries out the spin adaptation of the equations. We evaluate the performance of our method both in terms of accuracy and efficiency compared to other widely used single- and multireference schemes for a range of closed- and open-shell systems.

This manuscript is organized as follows: Section 2 first reviews the RIC-MRCC method and then presents the principles behind the spin-free formulation. Subsequently, Section 3 provides implementation details of the method in ORCA, including the translation layer that was developed between the two code generators Wick&d and AGE. In Section 4, numerical results are presented, including computational timings compared to state-of-the-art single- and multireference methods, accuracy on a benchmark of transition-metal ions and a large-scale calculation on the vitamin B_12_ model. In addition, the effect of the free parameter in the regularization factor on the stability and accuracy of the method is investigated. Finally, Section 5 concludes the paper with a summary of the main findings and an outlook for future work.

## 2 Theory

### 2.1 Generalized Normal-Ordering

The generalized normal-ordering (GNO) formalism developed by Mukherjee and Kutzelnigg^29,30^ extends the concept of normal-ordering to general multideterminantal vacuum states |Ψ_0_⟩. Although this framework is applicable to arbitrary wave functions, in this study, |Ψ_0_⟩ is assumed to originate from a complete active space (CAS) procedure^37,38^

1 $$|\Psi_{0}\rangle =\sum_{t=1}^{N_{CAS}}c_{t}|\phi_{t}\rangle$$

where *t* runs over the *N*_CAS_ many-body expansion functions, such as configuration state functions (CSF) or Slater determinants, |ϕ_*t*_⟩ in the active space. This manuscript relies on conventions for the orbital indices which are summarized in Table 1.

**Table 1:** Orbital Space Decomposition and Corresponding Index Conventions

space	symbol	indices	definition
internal	C	*i*, *j*	occupied
active	A	*t*, *u*, *v*, *x*, *y*, *z*	active
virtual	V	*a*, *b*	unoccupied
hole	H	*k*, *l*, *m*, *n*	H=C∪A
particle	P	*c*, *d*, *e*, *f*	P=A∪V
general	G	*p*, *q*, *r*, *s*	G=H∪V

Adopting the terse notation for second-quantized operators in spin–orbital basis

2 $${â}_{rs...}^{pq...}={â}_{p}^{†}{â}_{q}^{†}...{â}_{s}{â}_{r}$$

the contractions arising from the generalized Wick’s theorem^29,30,39^ yield the one-particle reduced density matrix (RDM) $\gamma_{u}^{t}=\left\langle \Psi_{0}\left|{â}_{u}^{t}\right|\Psi_{0}\right\rangle$ and the one-hole density matrix η_*u*_^*t*^ = δ_*u*_^*t*^ – γ_*u*_^*t*^, along with multilegged contractions producing *n*-body density cumulants,^40^ which are composed of antisymmetrized products of *n*- and lower-body RDMs. For the two-body case, it takes the form

3 $$\lambda_{vx}^{tu}=\gamma_{vx}^{tu}-\gamma_{v}^{t}\gamma_{x}^{u}+\gamma_{x}^{t}\gamma_{u}^{u}$$

A concise summary of the contractions rules arising from the GNO formalism can be found in ref 35, while the original publications by Mukherjee and Kutzelnigg^29,30^ provide a thorough discussion.

On this basis, the Born–Oppenheimer electronic Hamiltonian can be expressed in normal-ordered form with respect to |Ψ_0_⟩ as

4 $$Ĥ=E_{0}+\sum_{pq}^{G}f_{q}^{p}\left\{{â}_{q}^{p}\right\}+\frac{1}{4}\sum_{pqrs}^{G}v_{rs}^{pq}\left\{{â}_{rs}^{pq}\right\}$$

where {·} denotes normal-ordered operators. The scalar term comprises the reference energy $E_{0}=\left\langle \Psi_{0}\left|Ĥ\right|\Psi_{0}\right\rangle$ , and the one-electron part is given by the generalized Fock matrix^41^

5 $$f_{q}^{p}=h_{q}^{p}+\sum_{m}^{C}v_{qm\;}^{pm}+\sum_{tu}^{A}v_{qu}^{pt}\gamma_{u}^{t}$$

Here, $h_{q}^{p}=\left\langle \phi_{p}\left|ĥ\right|\phi_{q}\right\rangle$ and $v_{rs}^{pq}=\left\langle pq∥rs\right\rangle$ correspond to the standard one-electron and antisymmetrized two-electron integrals (in ⟨12|12⟩ physics notation), respectively.

### 2.2 Renormalized Internally-Contracted Multireference Coupled Cluster Theory

The renormalized internally contracted multireference coupled cluster theory^21^ (RIC-MRCC) introduced by some of the authors of this work is a natural extension of Evangelista’s driven similarity renormalization group (DSRG) theory^18–20^ with the key difference of relying on a nonunitary similarity transformations, thereby resembling internally contracted multireference coupled cluster (IC-MRCC)^5,22–28^ schemes. Instead of repeating the derivation of this theory starting from DSRG—as presented in the original publication^21^—this work relates the working equations directly to the ones from IC-MRCC.

In IC-MRCC, the wave function ansatz is defined by applying the cluster operator $e^{\mathrm{T̂}}$ to the reference CAS solution

6 $$|\Psi_{IC‐MRCC}\rangle =e^{\mathrm{T̂}}|\Psi_{0}\rangle$$

The cluster operator is decomposed in terms of *n*-body excitation operators, typically truncated at a chosen excitation rank. In the initial work on RIC-MRCC,^21^ a perturbative triples correction was introduced—based on the work of Hanauer and Köhn^10^—on top of the iterative singles and doubles solution. This extension, however, lies beyond the scope of the present study and will be addressed elsewhere. Hence, in this work, T̂ will be restricted to single and double excitations from the hole (H) to the particle (P) space

7 $$\mathrm{T̂}={\mathrm{T̂}}_{1}+{\mathrm{T̂}}_{2}=\sum_{k}^{H}\sum_{c}^{P}t_{k}^{c}\left\{{â}_{k}^{c}\right\}+\frac{1}{4}\sum_{kl}^{H}\sum_{cd}^{P}t_{kl}^{cd}\left\{{â}_{kl}^{cd}\right\}$$

Excitations involving only active indices are omitted from the cluster operator as they would lead to nontruncating coupled cluster equations.^5^ Their influence is confined to modifying the CAS expansion coefficients and can therefore be regarded as a reference relaxation effect. In IC-MRCC and DSRG schemes, this phenomenon is typically accounted for by solving the CAS problem for the effective Hamiltonian H̅,^5,28^ an approach that we intend to incorporate in future work. The effective Hamiltonian is obtained by similarity transformation of the Born–Oppenheimer Hamiltonian by the cluster operator

8 $$\mathrm{H̅}=e^{-\mathrm{T̂}}Ĥe^{\mathrm{T̂}}$$

In conventional CC theory, the energy *E*_CC_ and the singles *r*_k_^c^ and doubles *r*_kl_^cd^ residual equations are derived by projecting the Schrödinger equation

9 $$\mathrm{H̅}|\Psi_{0}\rangle =E_{CC}|\Psi_{0}\rangle$$

onto the reference and internally contracted excited configurations, respectively

10 $$E_{CC}≔\left\langle \Psi_{0}\left|\mathrm{H̅}\right|\Psi_{0}\right\rangle$$

11 $$r_{k}^{c}≔\left\langle \Psi_{0}\left|{â}_{c}^{k}\mathrm{H̅}\right|\Psi_{0}\right\rangle \overset{!}{=}0$$

12 $$r_{kl}^{cd}≔\left\langle \Psi_{0}\left|{â}_{cd}^{kl}\mathrm{H̅}\right|\Psi_{0}\right\rangle \overset{!}{=}0$$

Note, however, that the excited configurations ${â}_{k}^{c}|\Psi_{0}\rangle$ and ${â}_{kl}^{cd}|\Psi_{0}\rangle$ are, in general, linearly dependent, which often results in numerical instabilities during the iterative optimization procedure. This is an issue inherent to the IC ansatz and is typically addressed in MR configuration interaction and perturbation theories by defining a linearly independent set of excitation operators through canonical orthogonalization.^2,31^ Careful treatment of this redundancy—particularly for the single particle excitations—is necessary in IC-MRCC to ensure orbital-invariance^28^ and size-extensivity^5,42^ of the method, aspects that have been the subject of thorough study in the literature.^14,32,33^ In addition, these schemes typically employ thresholds for discarding linearly dependent terms, which may introduce discontinuities in the energies along a potential energy surface.^15^

An alternative approach for defining the residual equations relies on expanding H̅ in terms of contributions grouped by *n*-body operators

13 $$\mathrm{H̅}={\mathrm{H̅}}_{0}+\sum_{pq}^{G}{\mathrm{H̅}}_{q}^{p}\left\{{â}_{q}^{p}\right\}+\frac{1}{4}\sum_{pqrs}^{G}{\mathrm{H̅}}_{rs}^{pq}\left\{{â}_{rs}^{pq}\right\}+...$$

Here, the energy is provided by the zeroth-order term—as all other terms contain normal-ordered operators which vanish when evaluating reference expectation values—while the singles and doubles residuals correspond to the effective one- and two-body components

14 $$E_{CC}≔{\mathrm{H̅}}_{0}$$

15 $$r_{k}^{c}≔{\mathrm{H̅}}_{k}^{c}\overset{!}{=}0$$

16 $$r_{kl}^{cd}≔{\mathrm{H̅}}_{kl}^{cd}\overset{!}{=}0$$

yielding the many-body residuals, a term coined in ref 16. The RIC-MRCC method relies on this form of the residuals and the working equations are derived using the GNO formalism. For single-determinantal reference wave functions, the projected and many-body residual formulations lead to identical equations.^1^ This equivalence does not hold, however, for multireference wave functions, where the many-body residual equations form a simpler set of equations than the projected ones.^16,43^ An additional advantage of this formalism is that the resulting residuals are devoid of any redundancy even for linearly dependent amplitudes.^16^

To obtain the working equations, H̅ is expanded according to the Baker–Campbell–Hausdorff (BCH) formula as

17 $$\mathrm{H̅}=Ĥ+\left[Ĥ,\mathrm{T̂}\right]+\frac{1}{2}\left[\left[Ĥ,\mathrm{T̂}\right],\mathrm{T̂}\right]+...$$

which we truncate at the 2-fold commutator, an approximation that has been shown to have a negligible effect on the accuracy of IC-MRCC methods.^5,28^ Even with this truncation, however, evaluating all the resulting contractions becomes impractical for anything beyond small, few-electron systems. Therefore, RIC-MRCC employs a set of additional approximations that neglect costly contractions involving amplitudes with active orbital indices from the 2-fold commutator [[Ĥ,T̂],T̂] . As in ref 15, distinct approximations are applied for the energy and residual equations:

- Energy contribution (*E*_CC_): contractions involving multiple amplitudes with three active indices are omitted.
- Residual contribution (*r_k_^c^, *r*_kl_^cd^*): contractions involving multiple amplitudes with active indices, as well as all those containing the two-body cumulant, are neglected.

Although these approximations have primarily been chosen to decrease the computational cost of the method, physical motivations for them can be found in the initial publication on RIC-MRCC.^21^ An important consequence of these simplifications is that, unlike the untruncated equations that depend on up to four-body cumulants, RIC-MRCC requires only up to three-body cumulants, as all contractions involving the four-body cumulant are omitted through the scheme. This reduction is especially beneficial for systems with large active spaces, where evaluating higher-order RDMs—and their associated cumulants—constitutes the primary bottleneck both in terms of computational cost and memory usage.

The resulting coupled cluster equations are solved using a direct inversion of the iterative subspace^44–46^ accelerated quasi-newton iterative procedure

18 $$t_{\nu }\leftarrow t_{\nu }+\frac{r_{\nu }}{\Delta_{\nu }}$$

where the compound index ν encompasses particle (upper) and hole (lower) indices and the preconditioner is given by the generalized Møller–Plesset denominators Δ_ν_ ≔ Δ_*cd*..._^*kl*...^ ≔ ϵ_*k*_ + ϵ_*l*_ + ... – ϵ_*c*_ – ϵ_*d*_, corresponding to diagonal elements of the generalized Fock matrix.

Clearly, this iterative procedure can suffer from numerical instabilities^16,47^ in the case of small or vanishing denominators that cause the second term in eq 18 to diverge. It also introduces a free parameter—know as the flow—which offers two illuminating limits: at *s* = 0, all amplitudes are forced to vanish, leaving the CASSCF reference unchanged, while in the limit *s* → *∞*, the effect of the renormalization is eliminated and the update rule reduces to eq 18, recovering the standard many-body IC-MRCC equations. To mitigate this problem in RIC-MRCC, the amplitude update rule is augmented by a renormalization factor

19 $$t_{\nu }\leftarrow \left(t_{\nu }\Delta_{\nu }+r_{\nu }\right)\;\frac{1-e^{-s\Delta_{\nu }^{2}}}{\Delta_{\nu }}$$

This modification ensures that even for problematic vanishing denominators the updated amplitudes remain bounded. Originating from DSRG theory, such renormalization factors have also been incorporated into single- and multireference perturbation theories such as regularized MP2 ^48^ and CASPT2.^49^ In the context of CASPT2, it serves precisely the same role as the well-known real^50^ and imaginary^51^ shift parameters for mitigating intruder states.

A notable feature of our theory is that, stemming from DSRG, the regularization factor requires semicanonical orbitals—those that diagonalize the generalized Fock operator in the internal, active, and virtual spaces separately. The method can, in principle, be made orbital-invariant at the expense of spoiling the simple structure of the regularization factor in eq 19.^52^ As a state-specific approach, this orbital canonicalization must therefore be performed individually for each electronic state of interest.

### 2.3 Deriving Spin-Free Equations

Our earlier work^21^ implemented the working equations for the RIC-MRCC method in a spin–orbital basis. In this work, these equations are reformulated in spin-free form, following the procedure outlined in ref 16 for the many-body residual formulation of IC-MRCC. The core idea is to identify relations between the coefficients of different spin components of tensors, allowing the reduction of spin–orbital quantities to a single representative set of spin indices from which all other spin sectors can be recovered. In the past, this principle has been applied to derive certain spin-adapted single-reference CC schemes.^53–56^ More recently, this approach has found applications in the context of multireference methods where it has been used to derive both many-body^16^ and projected^33^ spin-free variants of IC-MRCC as well as DSRG equations.^57^

The next two subsections establish fundamental properties of general antisymmetric singlet tensors, which enables us to identify a minimal set of nonredundant spin–orbital components. These coefficients are subsequently expressed in terms of spin-free quantities, allowing the removal of all spin labels from the tensors present in the contractions. Then, we demonstrate that all tensors involved in the contractions can be indeed considered as singlets, thereby justifying their replacement by their spin-free counterparts. Our derivation closely follows the treatment given in the appendix of ref 58.

#### 2.3.1 Singlet Constraining Conditions for Antisymmetric Tensors

Consider a general *n*-body operator

20 $$Ô=\sum_{pq...}\sum_{\begin{array}{l} \sigma_{1}\sigma_{2}...\\ \tau_{1}\tau_{2}... \end{array}}^{\left\{\alpha ,\beta \right\}}\;o_{r_{\tau_{1}}s_{\tau_{2}...}}^{p_{\sigma_{1}}q_{\sigma_{2}}...}\;{â}_{r_{\tau_{1}}s_{\tau_{2}}...}^{p_{\sigma_{1}}q_{\sigma_{2}}...}$$

with operators ${â}_{r_{\tau_{1}}s_{\tau_{2}}...}^{p_{\sigma_{1}}q_{\sigma_{2}}...}$ and corresponding expansion coefficients *o*_*r*_τ1_ *s*_τ2_..._^*p*_σ1_ *q*_σ2_...^. The indices {*p*, *q*, ...} identify spatial orbitals and {σ_1_, σ_2_, ...}, {τ_1_, τ_2_...} label α and β spin components, respectively. We adopt the convention that lowercase indices refer to spin–orbitals, and uppercase indices to spatial orbitals. If a spin label is omitted from a spin–orbital index, the index is assumed to correspond to the α component while an overbar denotes the β component. Additionally, note that, within this subsection, the indices *t* and *u* refer to general indices instead of active ones.

The operator in eq 20 is considered antisymmetric if its coefficients *o*_*r*_τ1_ *s*_τ2_..._^*p*_σ1_ *q*_σ2_...^ are antisymmetric under permutations of either upper or lower indices

21 $$o_{r_{\tau_{1}}s_{\tau_{2}}...}^{p_{\sigma_{1}}q_{\sigma_{2}}...}=-o_{r_{\tau_{1}}s_{\tau_{2}}...}^{q_{\sigma_{2}}p_{\sigma_{1}}...}=-o_{s_{\tau_{2}}r_{\tau_{1}}...}^{p_{\sigma_{1}}q_{\sigma_{2}}...}=o_{s_{\tau_{2}}r_{\tau_{1}}...}^{q_{\sigma_{2}}p_{\sigma_{1}}...}$$

Additionally, for the tensor to constitute a singlet, it must commute with the three standard spin angular momentum operators $\left[{Ŝ}_{+},Ô\right]=\left[{Ŝ}_{-},Ô\right]=\left[{Ŝ}_{z},Ô\right]=0$ . By explicitly evaluating these commutators, one can derive singlet constraints,^58^ which force certain coefficients to vanish and relate coefficients of different spin sectors to each other. The key results of this procedure are

-
$\left[{Ŝ}_{z},Ô\right]=0$ implies that coefficients that do not conserve the *M*_S_ quantum number—coefficients where the number of α and β indices in the upper and lower sets of indices differ—must vanish.
- Evaluating $\left[{Ŝ}_{-},Ô\right]$ yields the following relations:

1. Coefficients where pairs of lower and upper α/β indices are exchanged are equivalent:
22 $$\begin{array}{llll} & 1‐body: & o_{q}^{p} & =o_{\mathrm{q̅}}^{\mathrm{p̅}},\\ & 2‐body: & o_{\mathrm{r̅}s}^{\mathrm{p̅}q} & =o_{r\mathrm{s̅}}^{p\mathrm{q̅}},\;o_{rs}^{pq}=o_{\overset{®}{rs}}^{\overset{®}{pq}},\\ & 3‐body: & o_{stu}^{pqr} & =o_{\overset{®}{stu}}^{\overset{®}{pqr}},\;o_{\overset{®}{st}u}^{\overset{®}{pq}r}=o_{st\mathrm{u̅}}^{pq\mathrm{r̅}} \end{array}$$
2. Coefficients containing only α spin indices can be expressed in terms of those containing a single β pair:
23 $$\begin{array}{llll} & 2‐body: & o_{rs}^{pq} & =o_{r\mathrm{s̅}}^{p\mathrm{q̅}}-o_{s\mathrm{r̅}}^{q\mathrm{p̅}},\\ & 3‐body: & o_{stu}^{pqr} & =o_{st\mathrm{u̅}}^{pq\mathrm{r̅}}-o_{st\mathrm{u̅}}^{\mathrm{pr}\mathrm{q̅}}-o_{st\mathrm{u̅}}^{rq\mathrm{p̅}} \end{array}$$
  Owing to the relation between the first and last coefficients in eq 21, these properties hold when permuting the upper indices, as shown in eq 23, as well as when permuting the lower indices.

These relations demonstrate that for one-, two-, and three-body operators, there exists only a single unique spin pattern—*o*_q_^p^, $o_{r\mathrm{s̅}}^{p\mathrm{q̅}}$ , and $o_{st\mathrm{u̅}}^{pq\mathrm{r̅}}$ , respectively—from which all other spin components can be obtained. Taking this into account dramatically reduces the number of nonredundant equations present in the spin–orbital form of the contractions. Note that these relations are entirely general, with no assumptions made on the nature of the tensor beyond its antisymmetry and singlet property.

Having identified a list of nonredundant spin–orbital quantities, the goal now is to express them in terms of spin-free quantities. The corresponding spin-free tensor coefficients *O*_*RS*..._^*PQ*...^ can be obtained by integrating out the spin degrees of freedom^59^

24 $$Ô=\sum_{PQRS...}O_{RS...}^{PQ...}\;{Ê}_{RS...}^{PQ...}$$

with

25 $${Ê}_{RS...}^{PQ...}=\sum_{\begin{array}{l} \sigma_{1}\sigma_{2}...\\ \tau_{1}\tau_{2}... \end{array}}^{\left\{\alpha ,\beta \right\}}\;{â}_{r_{\tau_{1}}s_{\tau_{2}}...}^{p_{\sigma_{1}}q_{\sigma_{2}}...}$$

corresponding to the standard spin-free excitation operators.^41^

Note that unlike spin–orbital coefficients, spin-free quantities are not antisymmetric under arbitrary index permutations; they are only symmetric with respect to simultaneous permutations of pairs of lower and corresponding upper indices comprising a column of indices

26 $$O_{RS...}^{PQ...}=O_{SR...}^{QP...}$$

The all-α component can be obtained by applying the antisymmetrizer of the symmetric group *S*_N_ to the spin-free tensor

27 $$o_{pq...}^{rs...}=\frac{1}{\left(N+1\right)!}\sum_{P\in S_{N}}{\;\left(-1\right)}^{\sigma }O_{RS...}^{P\left(PQ...\right)}$$

where *N* is the number of upper (or lower) indices and σ corresponds to the parity of the permutation P .^58^ For example, for one-, two- and three-body operators, this yields the following relations

28 $$o_{r}^{p}=\frac{1}{2}O_{R}^{P}$$

29 $$o_{rs}^{pq}=\frac{1}{6}\;\left(O_{RS}^{PQ}-O_{RS}^{QP}\right)$$

30 $$o_{stu}^{pqr}=\frac{1}{24}\;(O_{\mathrm{STU}}^{\mathrm{PQR}}-O_{\mathrm{STU}}^{\mathrm{PRQ}}-O_{\mathrm{STU}}^{QPR}-O_{\mathrm{STU}}^{RQP}+O_{\mathrm{STU}}^{RPQ}+O_{\mathrm{STU}}^{QRP})$$

By exploiting the property

31 $$O_{STU}^{PQR}+{\;O}_{STU}^{PRQ}+{\;O}_{STU\;}^{QPR}+O_{STU}^{RQP}+O_{STU}^{RPQ}+O_{STU}^{QRP}=0$$

valid for RDMs and cumulants,^58^ the expression for the three-body operator can be simplified further

32 $$o_{stu}^{pqr}=\frac{1}{12}\left(O_{STU}^{PQR}+O_{STU}^{RPQ}+O_{STU}^{QRP}\right)$$

Recall that, as demonstrated by eq 23, these all-α quantities are redundant and can be expressed in term mixed αβ spin components. To express only the nonredundant components, partial-trace relations^58^ can be exploited, which state that integrating out a subset of the spin indices leaves the remaining spin–orbital coefficients satisfying the same spin relations as lower-body operators. For instance, the partially traced operator containing a single pair of spin–orbital indices

33 $$O_{s_{\alpha }TU...}^{p_{\alpha }QR...}≔\sum_{\begin{array}{l} \sigma_{1}\sigma_{2}...\\ \tau_{1}\tau_{2}... \end{array}}^{\left\{\alpha ,\beta \right\}}o_{s_{\alpha }t_{\tau_{1}}u_{\tau_{2}}...}^{p_{\alpha }q_{\sigma_{1}}r_{\sigma_{2}}...}$$

satisfies $O_{s_{\alpha }TU...}^{p_{\alpha }QR...}=O_{s_{\beta }TU...}^{p_{\beta }QR...}$ , mirroring the one-body relation from eq 22. Using the partial trace relations, the nonredundant spin component can be expressed entirely in terms of spin-free quantities, as illustrated here for a two-body operator

34 $$o_{rs}^{pq}+o_{r\mathrm{s̅}}^{p\mathrm{q̅}}\overset{\left(33\right)}{≕}O_{rS}^{pQ}\overset{\left(28\right)}{=}\;\frac{1}{2}O_{RS}^{PQ}$$

35 $$↔o_{r\mathrm{s̅}}^{p\mathrm{q̅}}=\frac{\;1}{2}O_{RS}^{PQ}-o_{rs}^{pq}$$

36 $$\overset{\left(29\right)}{↔}o_{r\mathrm{s̅}}^{p\mathrm{q̅}}=\frac{1}{\;2}O_{RS}^{PQ}-\frac{1}{6}\left(O_{RS}^{PQ}-O_{SR}^{QP}\right)$$

37 $$↔o_{r\mathrm{s̅}}^{p\mathrm{q̅}}=\frac{1}{6}\left(2O_{RS}^{PQ}+O_{SR}^{QP}\right)$$

The same approach can be generalized to arbitrary *n*-body operators, using partial traces to connect nonredundant components to lower-body relations. Explicit formulas for up to four-body operators can be found in ref 58.

It should be stressed that all of the above relations are only formally valid for singlet operators. Therefore, to make use of these relations within our approach, it is necessary to demonstrate that all tensors involved in the contractions from the generalized Wick’s theorem are indeed singlets. These tensors consist of one- and two-electron integrals, cluster amplitudes, one-particle and one-hole RDMs, and higher-order cumulants. The integrals stem from a spin-adapted CAS self-consistent field (CASSCF) calculation that relies on a restricted set of molecular orbitals, guaranteeing therefore the singlet nature of the integrals. Since the MRCC wave function should not alter the spin of the reference CASSCF solution, the cluster amplitudes must act as singlets in order to conserve this property. The remaining question is why the RDMs and cumulants derived from a CASSCF wave function behave as singlets, even when the reference state can exhibit arbitrary spin multiplicity—an issue examined in the following section.

#### 2.3.2 Extensions to Spin Multiplets

In principle, the derivation presented so far is not immediately applicable to reference states with higher than singlet spin multiplicities. One reason for this restriction is that spin–orbital RDMs generally depend on the spin projection quantum number $M_{S}$ and, consequently, lack invariance under spin rotations. As they are defined in terms of expectation values, however, their spin-dependence can be simply integrated out as in eq 24. The resulting spin-free quantity is identical for all states within the multiplet,^40^ a property that can be demonstrated through the Wigner-Eckart theorem.^58^ Another reason is that, unlike RDMs, cumulants are not defined in terms of expectation values (see eq 3) and, therefore, performing simple spin summation yields quantities that remain $M_{S}$ -dependent.^40^ Several strategies have been proposed to eliminate this dependency.^40^ The approach adopted in this work relies on the observation that, for any spin multiplicity, an $M_{S}$ -invariant state can be constructed by forming an equally weighted ensemble average over all members of the multiplet

38 $$\rho \left(S\right)=\frac{1}{2S+1}\sum_{M_{S}=-S}^{S}|\Psi \left(S,M_{S}\right)\rangle \langle \Psi \left(S,M_{S}\right)|$$

resulting in a state that is a singlet. Here, S denotes the total spin quantum number. The spin- and spatial orbital RDMs can be extracted from ρ(S) by taking the trace

39 $$\gamma_{rs...}^{pq...}\left(S\right)=tr\left(\rho \left(S\right){â}_{rs...}^{pq...}\right)$$

40 $$\Gamma_{RS...}^{PQ...}=tr\left(\rho \left(S\right){Ê}_{RS...}^{PQ...}\right)$$

Performing spin integration of the density cumulants, using the spin–orbital RDMs defined in eq 39 results in expressions that are $M_{S}$ -independent as they can be written entirely in terms of spin-free RDMs from eq 40.^40,58,60^

One might assume that constructing the ensemble-averaged density matrix ρ(S) and corresponding RDMs requires solving for every state in the multiplet—a task that becomes increasingly expensive for higher multiplicities. For instance, in the context of DSRG, Li and Evengelista initially proposed to evaluate the ensemble-averaged RDMs by, first, computing the high-spin $M_{S}=S$ state and, then, successively applying the lowering operator ${Ŝ}_{-}$ to recover the lower spin states in the multiplet.^57^ Since the final spin-adapted equations only require spin-free RDMs, this procedure can be avoided by exploiting the linearity of the trace and recalling that any spin-independent quantity is identical, by definition, for each state in the multiplet and, hence, also for the ensemble-averaged state as a whole

$$tr\left(\rho \left(S\right){Ê}_{RS...}^{PQ...}\right)$$

41 $$=\frac{1}{2S+1}\sum_{M_{S}=-S}^{S}\;tr\left(|\Psi \left(S,M_{S}\right)\rangle \langle \Psi \left(S,M_{S}\right)|{Ê}_{RS...}^{PQ...}\right)$$

42 $$=\frac{1}{2S+1}\sum_{M_{S}=-S}^{S}\Gamma_{RS...}^{PQ...}=\Gamma_{RS...}^{PQ...}$$

Therefore, if the spin-free RDMs are available from a spin-adapted CAS solver, as in the case of the ORCA program,^34^ they can directly be used to compute the corresponding spin-free cumulants. Otherwise, if only a CAS solver in spin–orbital basis is available, it is sufficient to resolve one of the members of the multiplet and integrate out the spin degrees of freedom to obtain the corresponding spin-free RDMs.^59^ We note that this limitation of DSRG has been resolved in subsequent releases of the method within the Forte package.^61^

## 3 Computational Details

Our implementation of the spin-free RIC-MRCCSD equations employs a combination of automatic code generation tools. ORCA’s native code generator AGE^36^ is capable of deriving the projected form of the residual equations. Therefore, Evangelista’s Wick&d program^35^ is needed instead to produce the many-body residual equations in spin–orbital basis. These equations are then processed by AGE, which performs the spin adaptation and produces the C++ code for evaluating the contractions. In the process, AGE further optimizes the resulting equations by factorizing them into binary tensor contractions and identifying reusable intermediates across contractions.^36^

### 3.1 Wick&d to AGE Translator

Once Wick&d generates the equations in its internal representation, it can output the resulting tensor contractions in several formats, including the familiar NumPy^62^ optimized Einsum^63^ expressions, on which our pilot implementation relied on.^21^ In the present study, a local version of Wick&d has been extended to emit the tensor contractions directly in an AGE compatible syntax.

An important subtlety in using Wick&d is that it does not automatically enforce the antisymmetry of the residuals inherited from their corresponding amplitudes. Instead, it computes the nonsymmetric tensor *g*_*p*_σ1_ *q*_σ2__^*r*_σ3_ *s*_σ4_^, which accounts for all standard prefactors, after which the residual may be obtained by antisymmetrizing the indices pertaining to the same orbital space. For example, in the case of a two-hole two-particle (2h 2p) excitation, the contributions to the residual *r*_*i*_τ1_ *j*_τ2__^*a*_σ1_ *b*_σ2_^ should be antisymmetrized as

43 $$r_{i_{\tau_{1}}j_{\tau_{2}}}^{a_{\sigma_{1}}b_{\sigma_{2}}}\leftarrow g_{i_{\tau_{1}}j_{\tau_{2}}}^{a_{\sigma_{1}}b_{\sigma_{2}}}-g_{j_{\tau_{2}}i_{\tau_{1}}}^{a_{\sigma_{1}}b_{\sigma_{2}}}-g_{i_{\tau_{1}}j_{\tau_{2}}}^{b_{\sigma_{2}}a_{\sigma_{1}}}+g_{j_{\tau_{2}}i_{\tau_{1}}}^{b_{\sigma_{2}}a_{\sigma_{1}}}$$

This design is driven by efficiency consideration, since antisymmetrizing the single tensor *g*_*p*_σ1_ *q*_σ2__^*r*_σ3_ *s*_σ4_^ is usually computationally less expensive than repeatedly evaluating the contractions with permuted indices in order to recover the other three contributions. Our spin-adapted implementation, however, only requires the mixed αβ spin sectors for two particle excitations. All of these contributions are then directly accumulated into the residual ri j̅ab̅ .

### 3.2 Excitation Classes

In ORCA, all tensors involved in the contractions—including amplitudes—are decomposed by excitation class, defined by the orbital spaces of the indices. For single-particle excitations, this results in three distinct classes with corresponding amplitudes *t*_I_^A^ (as in single-reference schemes), *t*_I_^T^, and *t*_T_^A^. For the doubles, the excitations fall into eight classes, analogous to the first-order interacting spaces in conventional IC-MR schemes.^64^ Figure 1 depicts all the distinct amplitude excitation classes.

**Figure 1:**
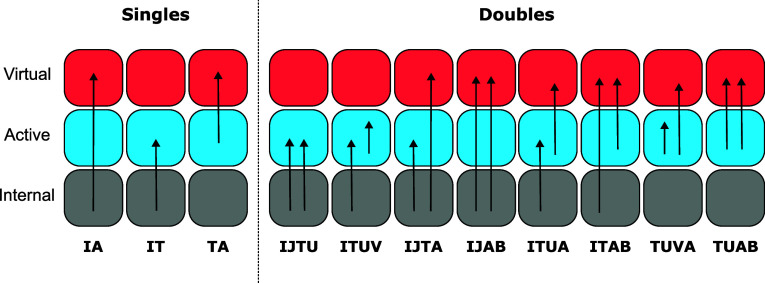
Breakdown of the amplitude excitation classes present in the RIC-MRCCSD scheme. Each column corresponds to a specific excitation class with each arrow representing a single-electron excitation. The name of the excitation class is found under each excitation with the first (two) and last (two) letters denoting the hole and particle spaces for the singles (doubles) excitations, respectively.

Each excitation class has a designated tensor that stores the coefficients for that class. To apply the singlet-constraining relations for doubles excitation that relate, for instance, $t_{r_{\alpha }s_{\alpha }}^{p_{\alpha }q_{\alpha }}$ to $t_{r_{\alpha }s_{\beta }}^{p_{\alpha }q_{\beta }}$ components, one must permute either the upper or lower indices of the tensor. In principle, either choice is valid. However, for some of the excitation classes, the two particle and hole spaces might differ from one another. For instance, the *t*_IJ_^TA^ tensor stores the coefficients corresponding to the excitation of two electrons from the internal space into an active and virtual orbital. In this case, the particle indices lie in different spaces, so their permutation in the tensor is not allowed as they index into different orbital spaces. The hole indices can safely be permuted—both being internal. Taking this into account, the following scheme is used in our implementation:

- For *t*_IT_^UV^ and *t*_IT_^AB^, the particle indices are permuted.
- For *t*_IJ_^JA^ and *t*_TU_^VA^, the hole indices are permuted.
- For *t*_IJ_^AB^, *t*_IJ_^TU^, and *t*_TU_^AB^, both particle and hole indices are in the same space, allowing either one to be permuted. We choose to permute the upper indices. Additionally, for these three classes, we exploit the permutational symmetry in eq 26 to halve the storage requirements of these tensors.
- The *t*_IT_^AU^ class is special: both hole and particle indices lie in different spaces, so neither set can be permuted. Therefore, for this class, we store both $t_{I\mathrm{T̅}}^{U\mathrm{A̅}}$ and $t_{I\mathrm{T̅}}^{\mathrm{U̅}A}$ in order to apply the singlet constraining relation
44 $$t_{IT}^{UA}=t_{I\mathrm{T̅}}^{U\mathrm{A̅}}+t_{I\mathrm{T̅}}^{\mathrm{U̅}A}$$

Note that these considerations are only needed for the amplitude tensors and not for the cumulants, since their indices lie entirely in the active space and can therefore be freely permuted. These singlet constraining relations alongside their redundant and nonredundant spin components for the excitation classes are summarized in Table 2.

**Table 2:** Singlet-Constraining Relations Summarizing Non-redundant Spin Components for the Amplitudes and Density Cumulants and Their Relations for Deriving Redundant Components^a^ ^b^ ^c^

class	non-redundant	relation
IA, IT, TA, TU	*o*_*q*_^*p*^	$o_{\mathrm{q̅}}^{\mathrm{p̅}}=o_{q}^{p}$
ITUV, ITAB, (IJAB, IJTU, TUAB, TUVW)	$o_{r\mathrm{s̅}}^{p\mathrm{q̅}}$	$o_{rs}^{pq}=o_{r\mathrm{s̅}}^{p\mathrm{q̅}}-o_{s\mathrm{r̅}}^{p\mathrm{q̅}}$
IJTA, TUVA, (IJAB, IJTU, TUAB, TUVW)	$o_{r\mathrm{s̅}}^{p\mathrm{q̅}}$	$o_{rs}^{pq}=o_{r\mathrm{s̅}}^{p\mathrm{q̅}}-o_{r\mathrm{s̅}}^{q\mathrm{p̅}}$
ITUA	$o_{r\mathrm{s̅}}^{p\mathrm{q̅}}$ , $o_{r\mathrm{s̅}}^{\mathrm{p̅}q}$	$$o_{rs}^{pq}=o_{r\mathrm{s̅}}^{p\mathrm{q̅}}+o_{r\mathrm{s̅}}^{\mathrm{p̅}q}$$
TUVXYZ	$o_{xy\mathrm{z̅}}^{tu\mathrm{v̅}}$	$o_{xyz}^{tuv}=o_{xy\mathrm{z̅}}^{tu\mathrm{v̅}}-o_{xy\mathrm{z̅}}^{tv\mathrm{u̅}}-o_{xy\mathrm{z̅}}^{vu\mathrm{t̅}}$

a Due to the decomposition of the tensors in terms of orbital spaces of the indices, only some of the relations can be used.

b Classes for which multiple rules can be applied are grouped in parentheses.

c The tensor *o* can symbolize amplitudes or density cumulants

### 3.3 Spin-Adaptation Procedure

With this theoretical framework in place, this section presents the complete procedure for spin adapting the many-body residual equations, with the overall workflow depicted in Figure 2.

**Figure 2:**
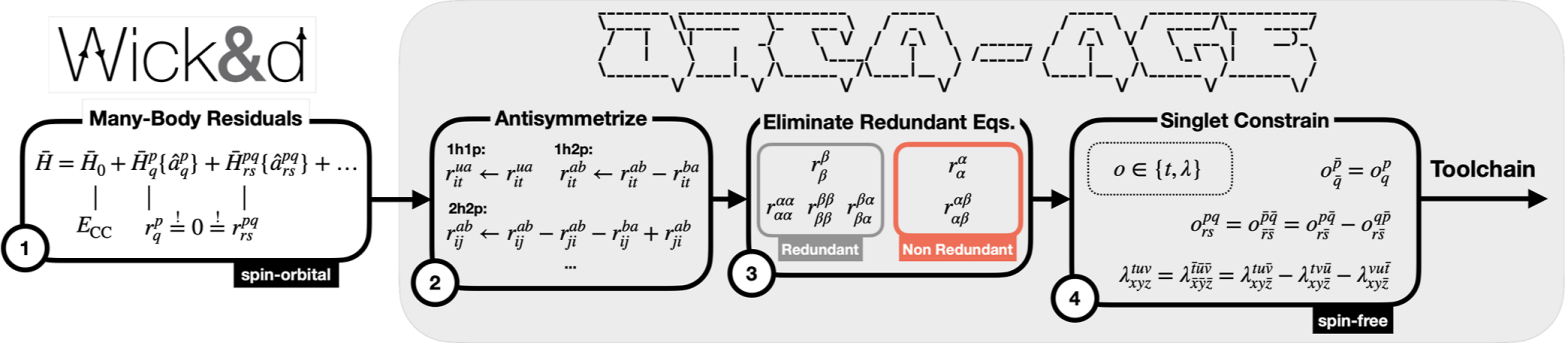
Procedure for deriving spin-free many-body residual equations. The pipeline begins (1) with spin–orbital residuals from Wick&d, (2) applies antisymmetrization, (3) discards redundant spin sectors and (4) exploits singlet constraints to produce nonredundant spin-free equations, which are then passed to the remaining AGE toolchain for C++ code generation. The notation *n*h*m*p in the antisymmetrization step (2) denotes the excitation class producing *n* holes and *m* particles.

Starting from the energy and residual equations produced by Wick&d in spin–orbital form, first, antisymmetrization is applied to the produced residuals as in eq 43. Then, explicit spin indices are introduced to the tensors, allowing residual equations pertaining to redundant spin sectors to be discarded, as these are not used to update nonredundant amplitudes. The tensors—namely the amplitudes and cumulants—involved in the remaining contractions are subsequently expressed in terms of nonredundant quantities via the singlet-constraining relations. Recall that these blocks are *o*_α_^α^, *o*_αβ_^αβ^, and *o*_ααβ_^ααβ^ for the one-, two-, and three-body operators, respectively. Each of these quantities can be cast in terms of their spin-free analogs, yielding equations in which spin dependence is entirely eliminated. These spin-free equations are then passed on to the remaining AGE toolchain, which optimizes the tensor contractions and generates high-performance, MPI-parallelized C++ code to execute them.

## 4 Results

In this section, we present a detailed benchmarking study of the RIC-MRCCSD scheme across a wide range of molecular systems. The primary goal is to validate key properties such as size consistency and to assess computational efficiency and accuracy in comparison to established electronic structure methods.

### 4.1 Computational Methodology

To evaluate the impact of spin adaptation, the execution times against both restricted and unrestricted single-reference coupled cluster methods are evaluated. These serve as the natural single-reference analogs. The accuracy and performance of RIC-MRCCSD are further assessed relative to prominent multireference approaches, including MR perturbation theory (MRPT), MR configuration interaction (MRCI), and MRCC—each expressed using the fully internally contracted formalism. Our MRPT calculations considered the widely used *n*-electron valence state perturbation theory of second order (NEVPT2)^3^ as well as a recent approximate fourth-order variant^65^ developed by some of the authors of the present work. In addition, the zeroth-order MR coupled-electron approximation method (CEPA(0)) with singles and doubles excitations is used as an approximation to the MRCISD equations, because it has demonstrated high accuracy in benchmark studies, frequently surpassing that of the latter.^15,66^ As the most accurate but computationally demanding method, the MRCCSD^36^) scheme implemented in ORCA was considered. This approach corresponds to the icMRCCSD-A scheme from the work of Hanauer and Köhn.^5^

Except for the NEVPT2 implementation, which was manually,^67^ all working equations for these methods were derived using the AGE automatic code generator from ORCA.^36^ All calculations were performed with a development version of ORCA 6.1.^34^ Unless stated otherwise, timing benchmarks were carried out on compute nodes equipped with two 12-core Intel­(R) Xeon­(R) E5-2687W v4 CPUs and 384 GiB of RAM.

### 4.2 Size Consistency

Due to the GNO, the RIC-MRCCSD equations are comprised solely of connected terms, which, in principle, assures the size-consistency of the method. In this section, this claim is verified numerically. A method will be considered size-consistent if the total energy of two noninteracting subsystems equals the sum of the energies of the individual subsystems. This property is critical for producing reliable energies across different molecular structures and is a key reason for the success of coupled cluster methods over configuration interaction approaches.

To probe size consistency, calculations were carried out on systems composed of three organic molecules—ethylene, butadiene, and hexatriene—using an active space corresponding to each polyene’s π-system and on the open-shell nitrogen and fluorine atoms. Combinations of these chemical species were placed 100 Ångström apart such that all interactions between them are eliminated. Then, the energy of the combined system was compared to the sum of the energies computed for each subsystem individually. For a size-consistent method, these energies should be identical. The size consistency error for a system, Δ*E*_sc_, is, hence given by

45 $$\Delta E_{sc}=E\left(\mathrm{supersystem}\right)+\sum_{i}E\left({\mathrm{subsystem}}_{i}\right)$$

Calculations were carried out with the def2-SVP^68^ atomic orbital basis set and tight convergence criteria, namely, 1 × 10^–12^ and 1 × 10^–9^ for the CASSCF energy and gradient, respectively, and 1 × 10^–9^ for the RIC-MRCCSD residuals, all in atomic units. Table 3 reports the size-consistency errors for both the CASSCF and RIC-MRCCSD solutions. All errors are within 10^–9^ *E*_h_ or smaller, with RIC-MRCCSD deviations comparable to those from CASSCF and of the same order of magnitude as the amplitude convergence criteria. Given that CASSCF is rigorously size-consistent, the small deviations observed can be attributed to numerical noise.

**Table 3:** Size Consistency Errors Reported in *E*_h_ for Combinations of Small Molecules and Atoms: ethylene, Butadiene, Hexatriene, N, and F

systems	Δ*E*_sc_ (CASSCF)	Δ*E*_sc_ (RIC-MRCCSD)
2 × ethylene	4.3 × 10^–11^	–1.58 × 10^–9^
2 × butadiene	7.4 × 10^–11^	–3.6 × 10^–11^
2 × hexatriene	2.9 × 10^–10^	–1.7 × 10^–9^
ethylene + butadiene	6.9 × 10^–11^	–5.2 × 10^–10^
ethylene + hexatriene	3.7 × 10^–11^	–1.1 × 10^–9^
2 × N	2.8 × 10^–13^	8.1 × 10^–12^
2 × F	6.5 × 10^–12^	6.7 × 10^–12^
3 × F	–1.0 × 10^–11^	–8.6 × 10^–10^

### 4.3 Comparison with Single-Reference Methods

This section assesses the efficiency of the spin-free implementation of the RIC-MRCCSD by benchmarking its performance against conventional single-reference coupled cluster schemes. For this comparison, the *trans*-stilbene molecule shown in Figure 3 was selected. This system features a fairly large active space of CAS­(14,14) comprised of the conjugated π-system spanning the two benzene rings and the two bridging carbon atoms.

**Figure 3:**
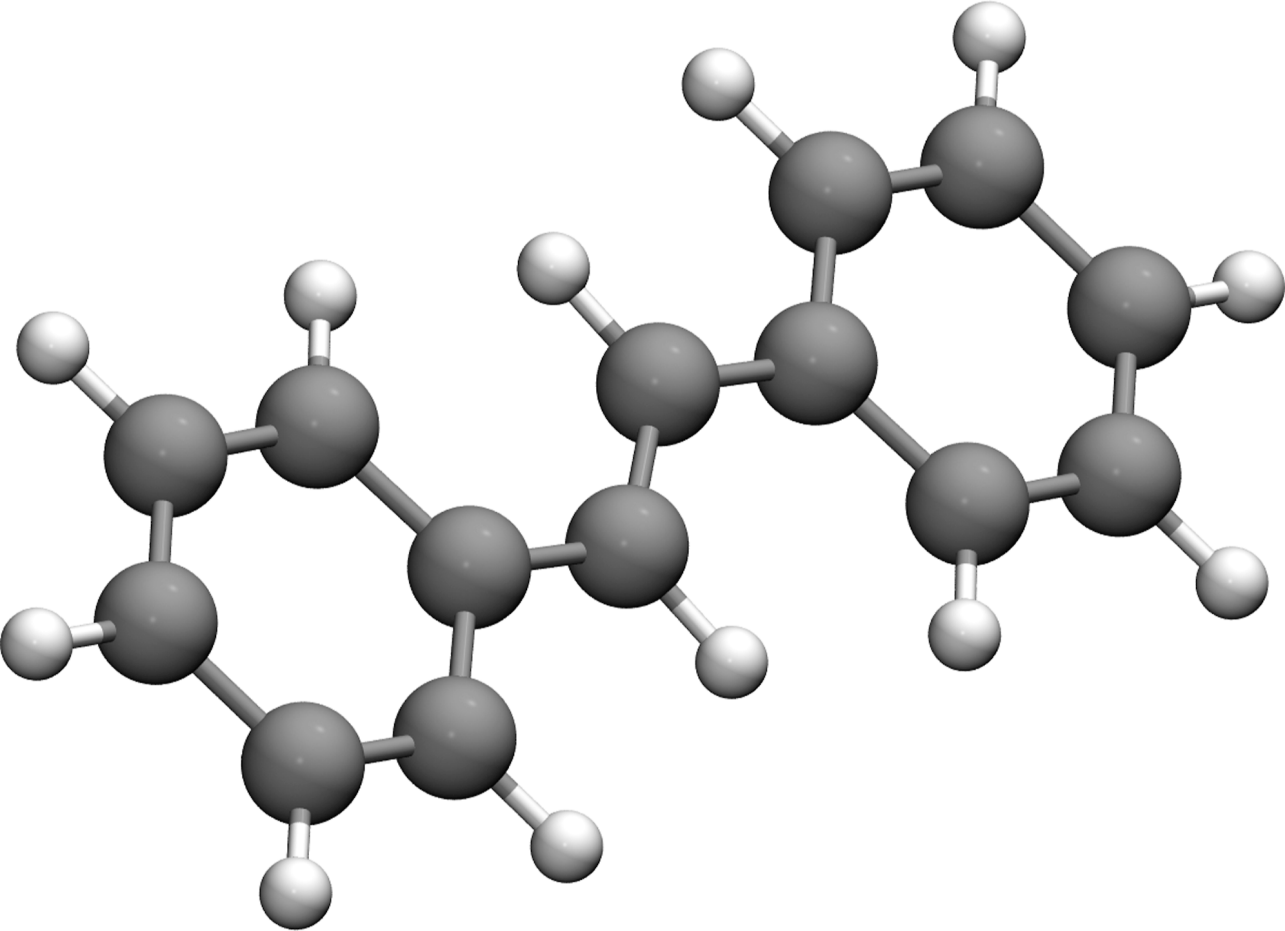
*Trans*-stilbene molecular structure (hydrogen atoms in white, carbon atoms in gray).

Single-iteration runtimes of the RIC-MRCCSD method are compared to those from both restricted and unrestricted SR-CCSD approaches. Before discussing the results, we briefly summarize the key differences in terms of the equations present in the two formalisms. SR schemes lack an active space containing partially occupied orbitals These orbitals have the particularity that electrons can be excited to and from—unlike the internal and virtual space. This leads to a huge number of additional equations in MR schemes, of which only a subset is preserved in RIC-MRCCSD for computational reasons (see above). In principle, the two methods share the contractions involving only internal and virtual indices. However, since RIC-MRCCSD truncates the BCH expansion at the second nested commutator, contractions arising from the third and fourth commutator—present in SR-CCSD—are omitted. Therefore, RIC-MRCCSD does not reduce to a regularized version of SR-CCSD in the limit of a vanishing active space (CAS­(0,0)).

To summarize, RIC-MRCCSD both introduces additional contractions due to the active space and omits certain contractions found in standard SR-CCSD. Nevertheless, after factorization, both approaches share the same rate-limiting contraction, as described in ref 21, and therefore exhibit the same formal computational scaling.

Table 4 summarizes the execution times for a single iteration of each coupled cluster method, covering both serial and parallel MPI runs with 2 to 16 processes on a single node. These data are based on calculations with the def2-TZVP^68^ basis set and the frozen-core approximation, keeping core orbitals doubly occupied throughout and thereby excluding excitations from these orbitals.

**Table 4:** Parallel Runtimes, in Seconds, of a Single Iteration of Various Coupled Cluster Methods for the *Trans*-stilbene Molecule in the def2-TZVP Bases^a^

	CCSD		
processes	RHF	UHF	RIC-MRCCSD
1	3590	18228	8580
2	2054	10240	5353
4	1224	6319	3711
8	838	4430	2145
16	743	3267	1869

a The RIC-MRCCSD scheme employs an active space of CAS­(14,14)

Our RIC-MRCCSD implementation demonstrates competitive performance compared to SR-CCSD methods. Each RIC-MRCCSD iteration is less than 3.5 times slower than RHF-CCSD, despite containing a significantly larger number of contractions. The data also highlight the efficiency gains from the spin adaptation as RIC-MRCCSD is substantially faster than the conventional unrestricted CCSD formalism. Figure 4 presents the timing data and the corresponding parallel efficiency

46 $$\mathrm{Parallel efficiency}=\frac{\mathrm{Speedup}}{\#\;\mathrm{Processes}}$$

as the number of MPI processes increases. Although parallel efficiency declines with the number of processes, significant speed-ups are still achieved, particularly with fewer processes. RIC-MRCCSD appears to exhibit slightly lower parallel efficiency with two and four processes, but overall, its scaling closely matches that of the single-reference schemes.

**Figure 4:**
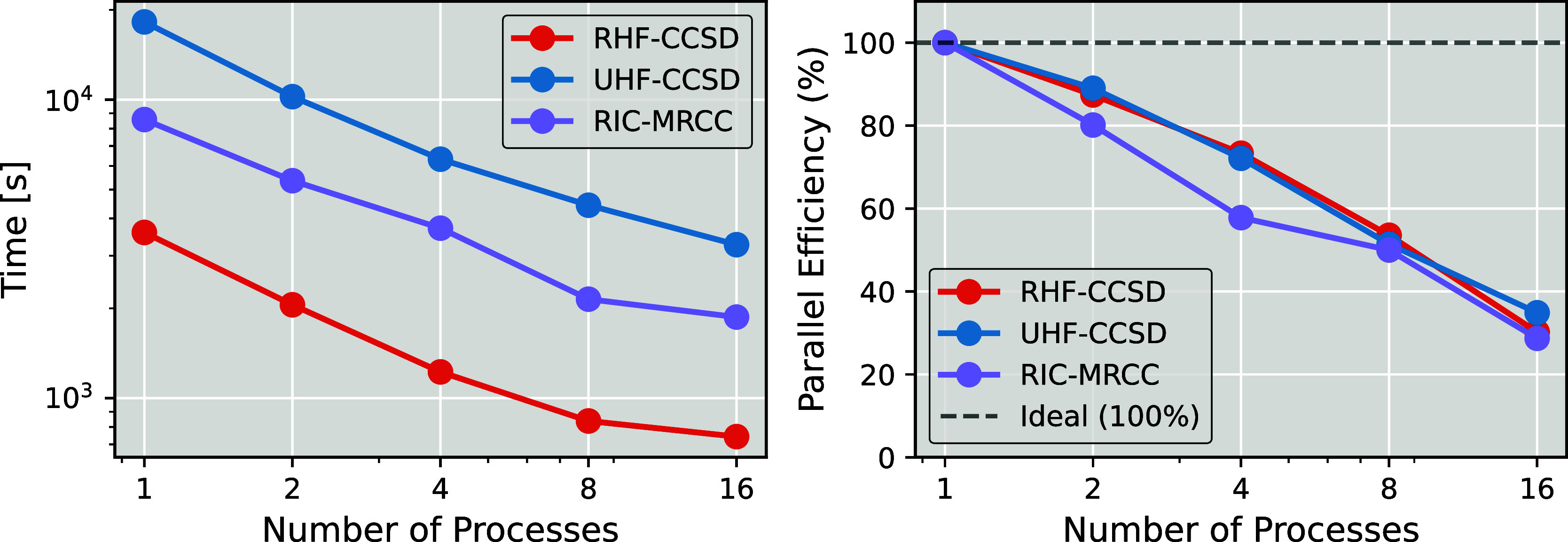
Execution time (left) and parallel efficiency (right) of a single iteration of various coupled cluster methods for *trans*-stilbene with a def2-TZVP basis and an active space of CAS­(14,14) for the RIC-MRCCSD method.

### 4.4 Scaling with Molecular Size

This section examines the computational scaling of the RIC-MRCCSD method using the all-*E* series of polyenes (2 to 14 carbon atoms) and corresponding active spaces with the def2-SVP^68^ basis set.

Figure 5 (left panel) shows the runtime per iteration for RIC-MRCCSD, CEPA(0), and NEVPT2 (which is assumed to converge in a single iteration), while the right panel reports the total execution times. Additionally, the number of iterations required for convergence and the fraction of the total runtime devoted to the RDM computations are indicated next to each bar. Initially, RIC-MRCCSD shows slower per-iteration runtimes than CEPA(0) for the smallest systems. However, this does not lead to longer total execution times, as RIC-MRCCSD consistently converges in 30% to 50% fewer iterations. In addition, RIC-MRCCSD shows significantly better scaling with active space size: for all polyenes larger than butadiene (CAS­(4,4)), each iteration is faster than CEPA(0), and the performance gap widens as system size increases. Moreover, the total runtime also scales more favorable with RIC-MRCCSD, as it does not require the evaluation of the 4-RDM. This tends to be a significant computational bottleneck in most schemes as illustrated by the CAS­(12,12) CEPA(0) calculation where almost half of the total runtime is spent on computing this quantity.

**Figure 5:**
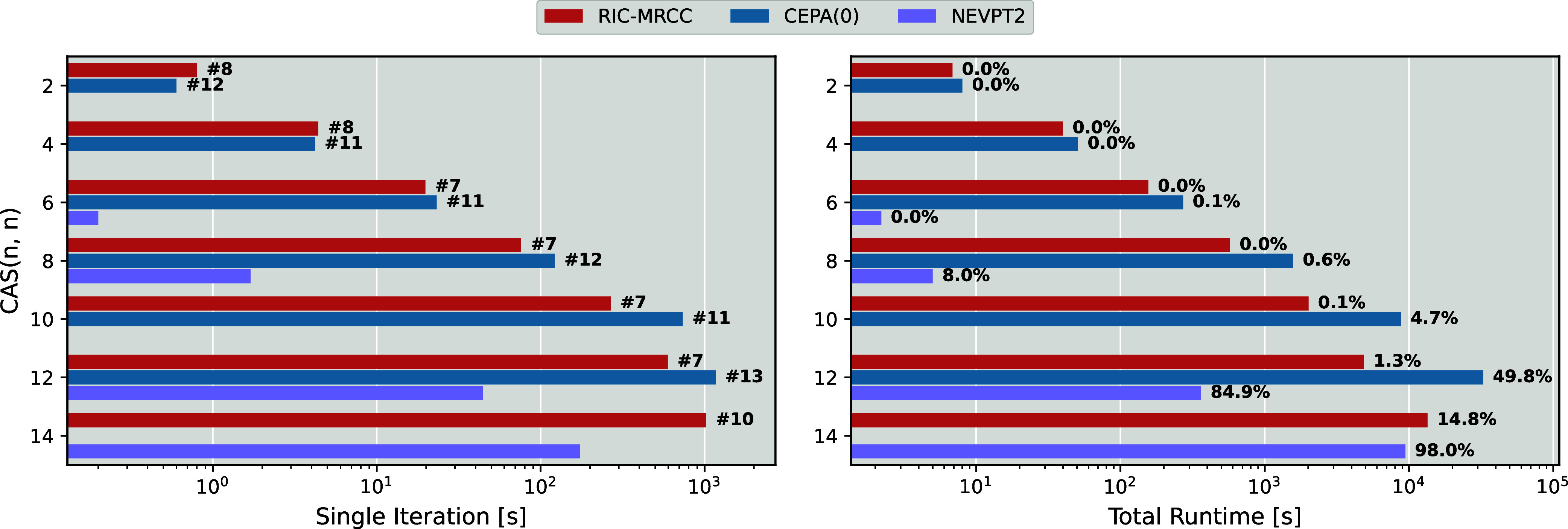
Runtimes for various multireference methods for an all-E polyene series (2 to 14 carbon atoms) with corresponding active spaces defined by the conjugated π-system. Left: timings per iteration with the number of iterations to convergence indicated next to each bar (except for NEVPT2 which is noniterative). Right: total runtimes for each method and the percentage of the total time taken for computing the 1-, 2- and 3-RDMs for RIC-MRCCSD (and the additional 4-RDM required for NEVPT2 and CEPA(0)). Note that for the largest polyene, CEPA(0) failed to converge and is therefore absent from the plots.

When comparing to NEVPT2 for the smaller systems, RIC-MRCCSD is not competitive in terms of time to solution, where NEVPT2 is substantially faster. It is encouraging, however, to see that once an active space size of CAS­(14,14) is reached, RIC-MRCCSD is only 40% slower than the highly optimized implementation of NEVPT2 in ORCA.^67,69^ Unlike CEPA(0), which evaluates the 4-RDM explicitly, the NEVPT2 implementation avoids this by directly computing the contribution of the 4-RDM to its equations.^67^ Despite this, this contribution still accounts for 98% of NEVPT2’s total runtime, which explains the narrowing performance gap between RIC-MRCCSD and NEVPT2 for larger active spaces.

### 4.5 Transition-Metal Ion Excitation Energies

This section assesses the accuracy of the RIC-MRCCSD method by comparing its state-averaged excitation energy errors to those from other multireference approaches, using a benchmark set of transition-metal ions.^70,71^ The benchmark consists of 56 excitation energies for seven divalent and seven trivalent fourth-row transition-metal ions, all calculated with a DKH-def2-QZVPP basis set.^68^ The energies are evaluated against experimental values found in the NIST database.^72^ These states are averaged over the *J* quantum number as outlined in the Supporting Information of ref 65, which also contains detailed electronic state assignments. The method was assessed on each system using a smaller active space containing only the 3*d* orbitals and a larger active space including also the 4*d* orbitals in order to account for the double-shell effect. For the copper ion, the 4*s* orbitals were also taken into the active space.

Note that our initial value of *s* = 0.5 *E*_*h*_^–2^ failed to converge with the larger active space for most states of the Co­(III), Cu­(III) and Ni­(III) metals. Such behavior was also observed by Li and Evangelista in their spin-free implementation of the sequentially transformed DSRG when studying iron-water and iron-ammonium clusters.^57^ Their solution was to decrease the flow parameter to *s* = 0.1 *E*_*h*_^–2^, which increases the regularization and should improve numerical robustness. This solution also resolved our convergence issues, with all calculations converging successfully for *s* ≤ 0.4 *E*_*h*_^–2^. It is important to note, however, that the RIC-MRCCSD iterations do not diverge for these systems. Instead, they converge exceedingly slowly as *s* is increased. For instance, the ground-state of Ni­(III) required 11, 18, 31, and 71 iterations to converge for *s* = 0.1, 0.2, 0.3 and 0.4 *E*_*h*_^–2^, respectively.

Figure 6 displays error distributions in excitation energies for both active space sizes. The left panel reports results from ref 65 for standard multireference methods while the right panel presents RIC-MRCCSD results for different flow parameter values. A breakdown of the errors for each electronic state can be found in the Supporting Information. The results in Figure 6 show that increasing the flow parameter *s* consistently reduces the error, as reflected in both the MAD and σ statistics. This trend is expected, since smaller values of *s* recover more dynamic correlation and, hence, generally improve accuracy, but at the expense of numerical robustness (as seen in the nonconvergent cases for *s* = 0.5 *E*_*h*_^–2^). For the largest value still permitting convergence (*s* = 0.4 *E*_*h*_^–2^), the method’s accuracy with the 3*d*-only active space is somewhere between that of NEVPT2 and NEVPT4. Including the 4*d* orbitals in the active space further reduces the error, with RIC-MRCCSD even outperforming IC-MRCC in terms of MAD. However, the conclusion from this observation is somewhat nuanced. The double-shell effect is traditionally invoked to compensate for missing dynamic correlation and should ideally not be necessary for high-accuracy MR methods. Indeed, this trend is observed for most of the standard MR methods, which barely change as the active space is expanded. As noted in ref 65, this is less conclusive for the NEVPT family of methods, where the error actually increases with the larger active space, suggesting that these methods benefit from some fortuitous error cancellation.

**Figure 6:**
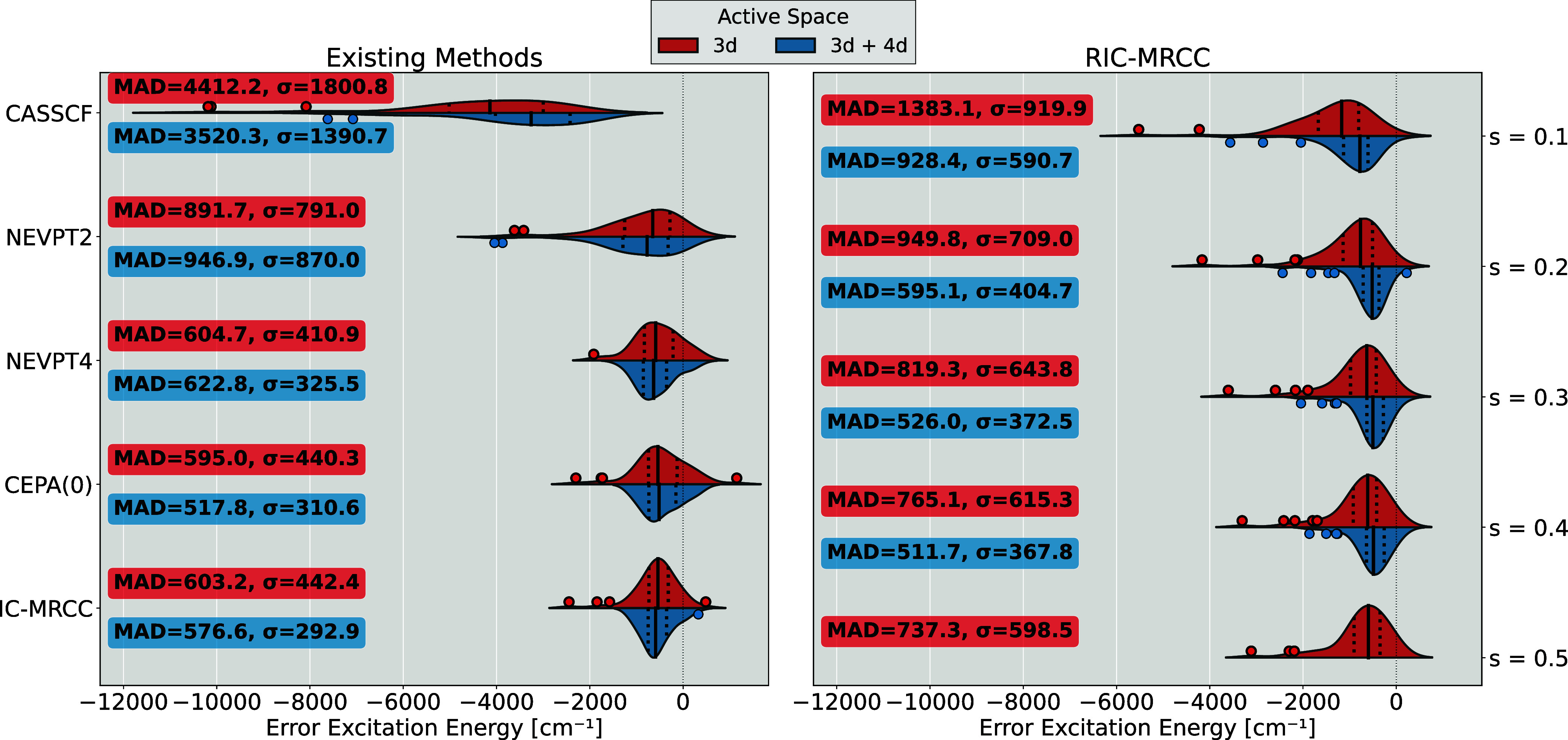
Errors in excitation energies relative to experimental data are shown for a benchmark set of 14 2+ and 3+ transition-metal ions. Results are evaluated using two active space sizes comprised of the 3*d* orbitals (red) and 3*d* + 4*d* orbitals (blue), shown as separate halves of a violin plot. Additionally, the solid line represents the mean, the dotted lines indicate the first and third quartiles, and outliers are also highlighted. The left plot reports data from ref 65 with IC-MRCC referring to the projection-based IC-MRCCSD formalism from Köhn, while the right plot presents new results for RIC-MRCCSD using various flow parameter values. Note that some RIC-MRCCSD calculations for the larger active space with *s* = 0.5 *E*_*h*_^–2^ did not converge and are omitted. Each plot is complemented by its mean absolute difference (MAD) and standard deviation (σ).

### 4.6 Ethylene Rotation

This section evaluates the accuracy of the RIC-MRCCSD method in comparison to established multireference approaches by examining the dihedral rotation of ethylene. The system is modeled using a CAS­(2,2) active space, consisting of the π-bonding and π-antibonding orbitals of the double bond using the def2-TZVP basis set. As the dihedral angle is rotated, the double bond is effectively broken and reformed near 90°.

To benchmark accuracy, we assess the errors of the RIC-MRCCSD method at various flow parameter values, alongside NEVPT at second, third, and fourth order, and CEPA(0), referencing the full IC-MRCC results. These findings are shown in Figure 7.

**Figure 7:**
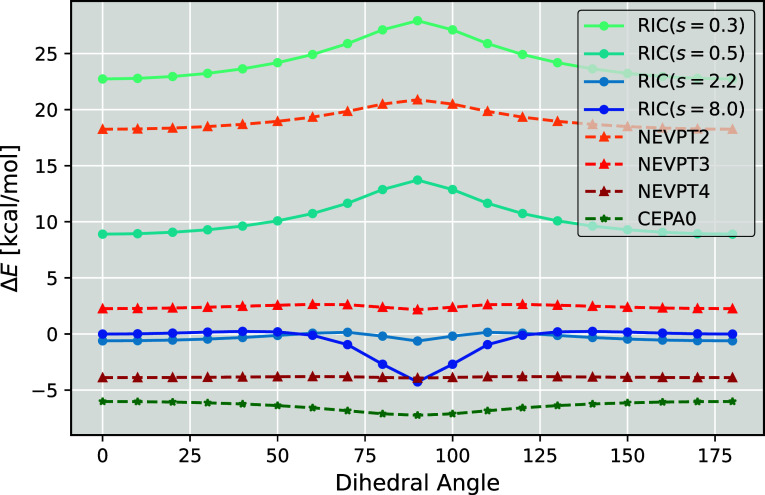
Error during dihedral angle rotation of ethylene evaluated relative to the IC-MRCCSD method for different multireference approaches.

A key observation is that the flow parameter significantly influences the errors in the RIC-MRCCSD method. Recall that the parameter *s* interpolates between the CASSCF solution at *s* = 0*E*_*h*_^–2^—lacking dynamic correlation—and the conventional many-body MRCC limit at *s* → ∞. Increasing *s* therefore enhances dynamic correlation capture, but can reduce numerical stability as observed in the previous section.

The behavior of RIC-MRCCSD curves for different flow parameters mirrors somewhat the one from the NEVPT sequence. At low perturbation order (NEVPT2 or *s* < 1 *E*_*h*_^–2^), both show a pronounced concave hump around the critical angle of 90°. As the order is increased and more dynamic correlation is recovered (NEVPT3 or *s* ≈ 2 *E*_*h*_^–2^), this feature is greatly reduced and, in fact, exhibits a convex profile. NEVPT4 becomes nearly flat, indicating consistent description of the correlation along the potential energy curve with respect to the IC-MRCCSD reference energies. In contrast, for RIC-MRCCSD, as *s* increases, the hump becomes progressively more prevalent and eventually, at *s* ≥ 12 *E*_*h*_^–2^, the method fails to converge. We suspect that this behavior is due to the emergence of intruder states as the regularization is diminished.

Although higher-order NEVPT methods yield excellent parallelity, their absolute energies do not converge to IC-MRCC—notably, NEVPT3 is closer than NEVPT4. Conversely, the RIC-MRCCSD scheme approaches IC-MRCCSD in absolute terms at large *s*, suggesting that the series of approximations involving the omission of expensive contractions are justified.

Evidently, the previously chosen value of *s* = 0.5 *E*_*h*_^–2^ in our initial study,^21^ as well as in related DSRG approaches, proves to be not optimal for ethylene. While *s* = 0.5 *E*_*h*_^–2^ performed well for diatomic molecules, these results highlight the empirical nature and selection challenges for this parameter. Note that in the context of DSRG, although the value of *s* = 0.5 *E*_*h*_^–2^ is considered a good general-purpose choice, certain studies have suggested optimal values ranging from 0.3 to 4.0 *E*_*h*_^–2^, depending on the particular variant of the method.^73^ To better assess the effect of the flow parameter on the incurred error of the potential energy curve along the dihedral angle, we report the nonparallelity error

47 NPE=max(ΔE)−min(ΔE)

which measures deviation from parallelity from the reference IC-MRCC curve. The NPE can be considered a more relevant metric than absolute energy differences, as constant energy offsets tend to cancel once observables and properties are computed.

Figure 8 displays the NPE across different values of *s*, alongside reference values from the other four previous dynamic correlation methods as well as the reference CASSCF value. The RIC-MRCCSD curves attain a local maximum around the value of *s* = 0.5 *E*_*h*_^–2^, after which it decreases until reaching the best NPE around *s* = 2.0 *E*_*h*_^–2^. Beyond this point, the NPE increases, likely due to intruder states, and ultimately the iterations fail to converge at *s* > 12 *E*_*h*_^–2^. Similar convergence ranges for the flow parameter are reported for MR-DSRG.^19,74^ The seemingly peculiar behavior at *s* < 0.5 *E*_*h*_^–2^, where the NPE seems to decrease as the flow is reduced, hence recovering less dynamic correlation, is attributable to the particularly parallel CASSCF reference, which is approached as *s* → 0 *E*_*h*_^–2^. Indeed, with a value of 1.63 kcal/mol, the CASSCF solution outperforms NEVPT2 in terms of NPE (2.63 kcal/mol), suggesting that low-order recovery of dynamic correlation can, in fact, degrade the parallelity of the reference wave function.

**Figure 8:**
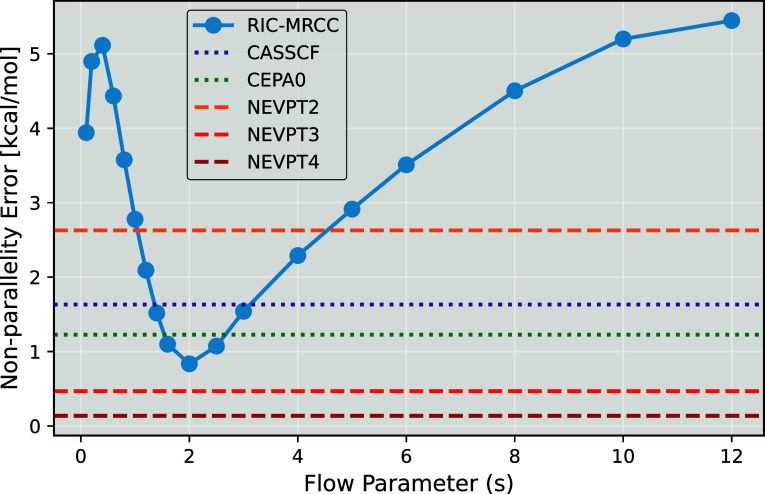
Ethylene nonparallelity error along a 180° dihedral rotation calculated for RIC-MRCCSD energies relative to the IC-MRCCSD curve as a function of the flow parameter *s*. Additionally, the CASSCF, CEPA(0) and NEVPT2, NEVPT3, and NEVPT4 NPEs are provided for comparison.

### 4.7 Size Stress Test: Vitamin B_12_

As a final benchmark to demonstrate the viability of the RIC-MRCCSD beyond small model systems, we report the execution time of the ground-state energy of a fairly large molecule. In particular, we study the vitamin B_12_ model from ref 75, where a simplified model of molecule, containing an additional histidine lower axial ligand, was constructed from high-resolution X-ray crystallographic data. The molecular structure of this model system is depicted in Figure 9.

**Figure 9:**
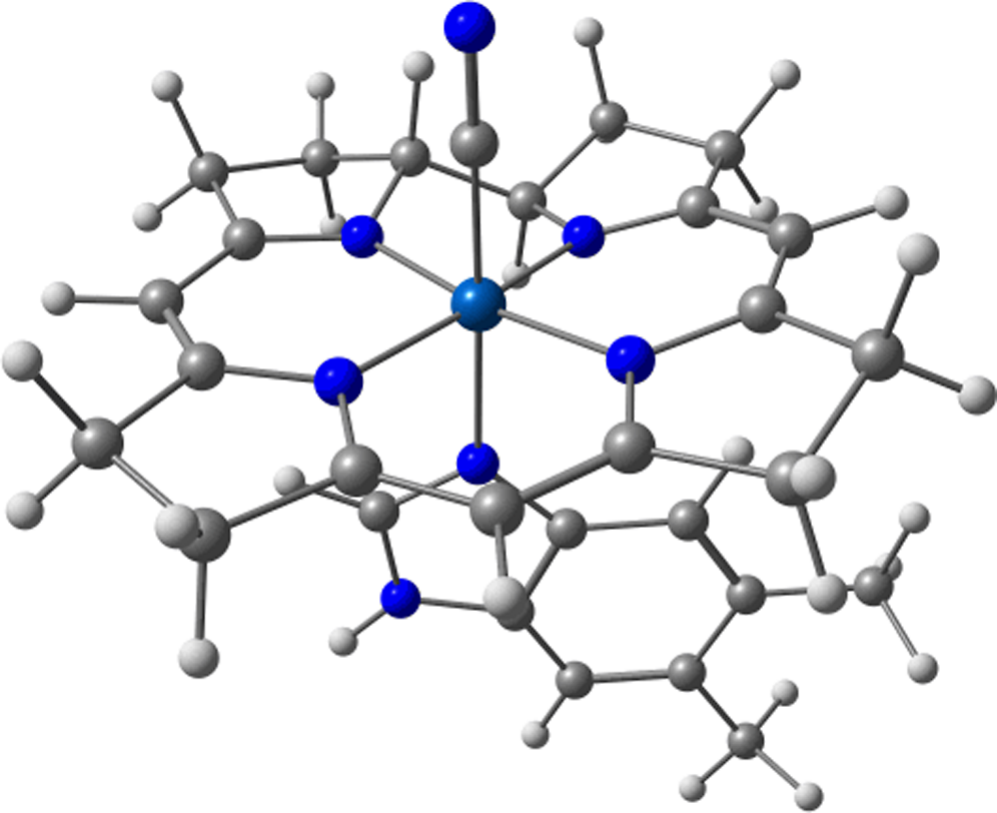
Vitamin B_12_ model system with its cobalt transition-metal center and corrin macrocycle augmented by a histidine lower axial ligand.

For selecting the active space of the molecule, we follow the procedure from the original study,^75^ which identified an active space of 12 electrons in 12 orbitals, which are illustrated in Figure 10.

**Figure 10:**
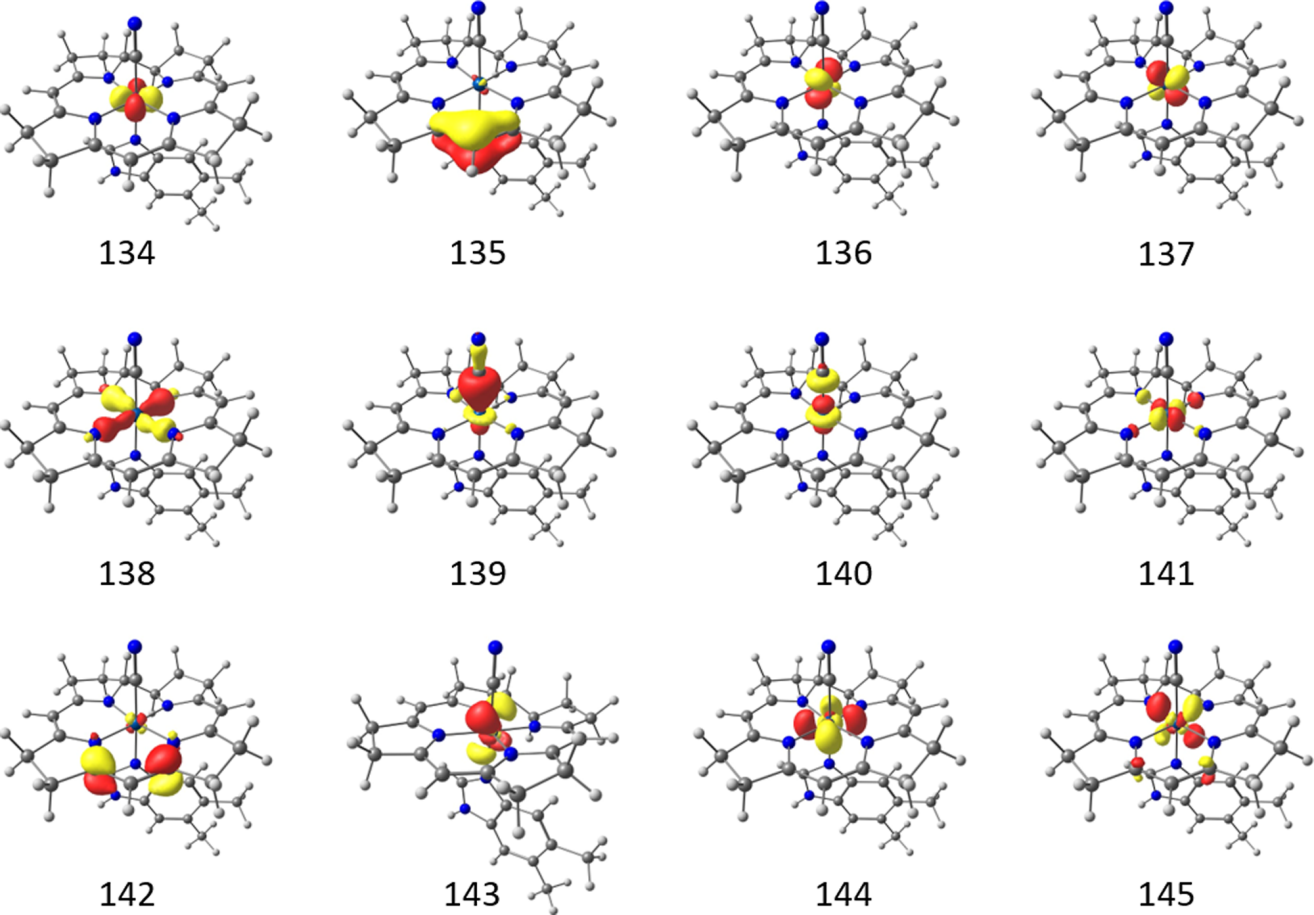
Vitamin B_12_ active space CAS­(12,12) comprised of the five 3*d* orbitals from the cobalt atom (134, 136, 137, 140, 141), an equatorial (138) and axial (139) bonding orbital, a π-bonding (135) and antibonding (142) pair from the corrin macrocycle and, finally, three additional 4*d* orbitals (143, 144 and 145) to account for the double-*d* shell effect.

As revealed by the original study, none of the excited states of the molecule maintain the character of the corresponding CASSCF solution, following the application of the dynamic correlation scheme, suggesting that state-specific methods, such as RIC-MRCCSD, would be unreliable for describing these states. Indeed, this state-mixing process was confirmed by us independently using the quasi-degenerate extension of the NEVPT2 method. For the ground-state, however, the CASSCF solution comprises 85% of the weight in the perturbed wave function, making it amenable state-specific schemes. Therefore, in this section, we restrict our focus to the computation of this single state.

Our calculations applied the x2c-TZVPall^76,77^ basis set for the first coordination sphere, while all other atoms were described with the smaller x2c-SVPall^76,77^ basis set to maintain computational feasibility. Using the frozen-core approximation, this setup yields 40 frozen, 94 internal, 12 active, and 663 virtual orbitals, representing a substantial system for high-accuracy multireference methods. We compare the performance of the RIC-MRCCSD schemes with SR RHF-CCSD and the NEVPT2 and NEVPT4 MR methods, each run on a 32-core AMD EPYC 75F3 processor with 16 parallel MPI processes. Runtimes and total memory usage for each approach are reported in Table 5.

**Table 5:** Performance Comparison for the Vitamin B_12_ Model

	method			
	NEVPT2	NEVPT4	RHF-CCSD	RIC-MRCCSD
time	446.3 [sec]	7.53 [hours]	3.49 [days]	3.87 [days]
memory [GB]	9.0	97.7	134.5	155.7

Although the CC methods do not match the efficiency of the NEVPT methods—particularly the highly optimized NEVPT2 implementation in ORCA—it is encouraging that the RIC-MRCCSD method requires only marginally more time and memory than conventional RHF-CCSD. This suggests that, with the present implementation, systems accessible to RHF-CCSD should also be accessible to RIC-MRCCSD.

## 5 Conclusions

In this work, we have introduced a spin-free formulation of the renormalized internally contracted multireference coupled cluster method with single and double excitations (RIC-MRCCSD) and present its efficient implementation within the ORCA quantum chemistry package. The implementation was accomplished by interfacing Evangelista’s Wick&d program—which generates the many-body residual equations in spin–orbital form—to ORCA’s native AGE code generator. The resulting equations are spin adapted by AGE through the use of singlet-constraining relations that relate different spin sectors of the spin–orbital quantities.

We have validated fundamental properties of the method, in particular size consistency, and assessed its overall performance on a set of molecular systems including organic compounds and transition metal ions and complexes. Our implementation showed comparable efficiency, both in terms of runtime and memory requirements, to the closed-shell single-reference coupled cluster module available in ORCA. Moreover, since the theory involves only up to three-body cumulants, the RIC-MRCCSD approach achieves competitive performance relative to the highly optimized NEVPT2 implementation when targeting large active spaces. As a demonstration of its applicability to extended systems, we computed the ground-state electronic energy of a vitamin B_12_ model comprising 809 basis functions and a CAS­(12,12).

With regard to accuracy, the method inherits a free parameter—the flow parameter *s*—from the closely related DSRG theory. This parameter governs not only the accuracy but also the numerical stability of the approach. Larger values of *s* recover a greater portion of the dynamic correlation but may also introduce intruder states. Such arbitrary parameters are common in multireference theories prone to intruder problems, with examples ranging from the shift parameter in CASPT2 to orthogonalization thresholds in IC-MRCC and the flow parameter in the closely related DSRG theory. Our benchmark results indicate a broad range of suitable values for this flow parameter. In the transition-metal ion calculations with enlarged active spaces including the double-*d* shell, a value of *s* = 0.4 *E*_*h*_^–2^ was required to ensure convergence across all electronic states of interest. However, for the organic molecule ethylene, this value appears too conservative, and a much larger choice of *s* = 2.2 *E*_*h*_^–2^ provides improved accuracy in terms of the nonparallelity of the potential energy curves.

This work represents an initial step toward incorporating theories based on the many-body residuals into the ORCA framework, laying the foundation for the development of related approaches. Future efforts will focus on analyzing the origin of the instabilities observed in the many-body formulation of IC-MRCC and on evaluating strategies for their mitigation. As observed in our pilot study^21^ and confirmed in this work, the RIC-MRCC method restricted to single and double excitations fails to consistently surpass the accuracy of established second-order multireference perturbation theories. To address this limitation, our earlier work also proposed a perturbative triples correction, RIC-MRCCSD­[T], which demonstrated strong potential in bridging this gap at moderate increase in computation cost and will, therefore, be the subject of further research.

## Supplementary Material



## Notes

The Supporting Information contains a description of the working equations for the IC-MRCC from Köhn, which was used as a reference for most of the benchmarks. Additionally, the molecular structure and energies are provided for all the calculations (PDF)
