# Supplementary material for: Efficient Implementation of the Spin-Free Renormalized Internally-Contracted Multireference Coupled Cluster Theory
Source: J Phys Chem A. 2026 Feb 3;130(6):1417–32. doi: 10.1021/acs.jpca.5c07588 (PMC12908158; doi:10.1021/acs.jpca.5c07588)
Supplement: Supplementary file 1 [file jp5c07588_si_001.pdf]

Supporting Information for Article: Efficient Implementation of the Spin-Free Renormalized Internally-Contracted Multireference Coupled Cluster Theory

Kalman Szenes,[†] Riya Kayal,[‡] Kantharuban Sivalingam,[‡] Robin Feldmann,[†] Frank Neese,[*,‡] and Markus Reiher[*,†]

†*ETH Zürich, Department of Chemistry and Applied Biosciences, Vladimir-Prelog-Weg 2, 8093 Zürich, Switzerland*

‡*Max-Planck-Institut für Kohlenforschung, Kaiser-Wilhelm-Platz 1, 45470 Mülheim an der Ruhr, Germany*

E-mail: neese@kofo.mpg.de; mreiher@ethz.ch

1 Orbital Index Convention

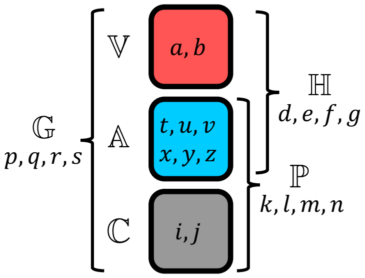

Figure S1: Orbital index convention followed throughout manuscript.

2 Fully Internally Contracted Multireference Coupled Cluster

The relation between a general internally contracted MRCC and the RIC-MRCC is layed out in Section 2.2. In the numerical evaluation, the ORCA implementation of the fully internally contracted multireference coupled cluster ansatz (FIC-MRCC) serves as a reference.[1] Hence, in this section, we recap key aspects of the method denoted as FIC-MRCC, which uses the same methodology as 'scheme A' in the seminal work of Hanauer and Köhn.[2] Here, the cluster operator

$$
\begin{aligned}
\hat{T} =& \frac{1}{2}\sum_{IJ}^{\mathbb{C}}\sum_{AB}^{\mathbb{V}} t_{IJ}^{AB}\hat{E}_I^A\hat{E}_J^B + \frac{1}{2}\sum_{IJ}^{\mathbb{C}}\sum_{TU}^{\mathbb{A}} t_{IJ}^{TU}\hat{E}_I^T\hat{E}_J^U \\
&+ \frac{1}{2}\sum_{TU}^{\mathbb{A}}\sum_{AB}^{\mathbb{V}} t_{TU}^{AB}\hat{E}_T^A\hat{E}_U^B + \sum_{I}^{\mathbb{C}}\sum_{AB}^{\mathbb{V}}\sum_{T}^{\mathbb{A}} t_{IT}^{AB}\hat{E}_I^A\hat{E}_T^B \\
&+ \sum_{IJ}^{\mathbb{C}}\sum_{A}^{\mathbb{V}}\sum_{T}^{\mathbb{A}} t_{IJ}^{AT}\hat{E}_I^A\hat{E}_J^T + \sum_{A}^{\mathbb{V}}\sum_{TUV}^{\mathbb{A}} t_{TU}^{AV}\hat{E}_T^A\hat{E}_U^V \\
&+ \sum_{I}^{\mathbb{C}}\sum_{A}^{\mathbb{V}}\sum_{TU}^{\mathbb{A}} \left(t_{IT}^{AU}\hat{E}_I^A\hat{E}_T^U + t_{IT}^{UA}\hat{E}_I^U\hat{E}_T^A \right) \\
&+ \sum_{I}^{\mathbb{C}}\sum_{TUV}^{\mathbb{A}} t_{IT}^{UV}\hat{E}_I^U\hat{E}_T^V,
\end{aligned} \tag{1}
$$

is directly defined in the spatial orbital basis using the spin-free excitation operators from Eq.(25).

The working equations are derived exploiting the fundamental commutation rules of the spin-free operators. Note that the double excitations in the cluster operator are identical to ones used in the FIC-CASPT2, FIC-NEVPT2 and FIC-MRCI theories,[3], where the Brillouin singles are implicitly included in the double excitations.

Following conventional coupled cluster theories, the amplitudes are obtained by solving projected residual equations

$$
r_{KL}^{CD} := \langle\Psi_0|\hat{E}_C^K\hat{E}_D^L\bar{H}|\Psi_0\rangle \stackrel{!}{=} 0, \tag{2}
$$

where $\hat{E}_K^C \hat{E}_L^D |\Psi_0\rangle$ correspond to internally contracted excited CSFs. In practice, the projection and subsequent amplitude update are carried out in the Dyall energy eigenbasis, which is orthonormal and linearly independent by construction.[3]

Both methods, RIC-MRCC and FIC-MRCC, keep the reference wavefunction $|\Psi_0\rangle$ fixed and truncate the similarity transformed Hamiltonian $\overline{H}$ at the second commutator of the BCH expansion. However, unlike the RIC-MRCC method, in FIC-MRCC all terms are retained without further approximations. The presence of the projection operator in Eq. (2) leads to expressions that require up to five-body RDMs, which severely limits its application to systems with larger active spaces. In contrast, the RIC-MRCC equations involve only up to three-body RDMs and cumulants.

3 Multiroot DIIS Solver

Starting with CASSCF or state-averaged CASSCF orbitals, the ground and excited states are obtained using a multiroot DIIS solver, which is sketched here:

1. Solve CAS-CI problem for a given multiplicity and a number of roots (NRoots).
2. Loop over roots=1,...,NRoots:
   (a) Compute the state-specific Fock operator and canonicalize the orbitals (including the actives).
   (b) Generate the full set of one- and two-electron integrals in molecular orbital basis.
   (c) Rotate the CAS-CI vector of the given root to match the orbital representation.[4]
   (d) Compute the reduced density matrices/cumulants up to rank 3.
   (e) Initialize RIC-MRCC amplitudes with a guess from second order Møller-Plesset perturbation theory.
   (f) Solve the residual equations for the given root using the DIIS.[5]

Key steps in the DIIS procedure are the computation of the residuals (Eq (10)-(12)) and the amplitude update (Eq (19)), which are described in the main article. The amplitudes are updated until the change of the largest residuals is less than the convergence threshold.

4 Size-Consistency Test

Table S1: Energies in E_h used to compute the size-consistency errors in the manuscript.

**Molecules**	E(CASSCF)	E(RIC-MRCCSD)
Ethylene	-78.005874853052	-78.274327708552
Butadiene	-154.864770066828	-155.375818408691
Hexatriene	-231.724283783962	-232.478561954799
2 × Ethylene	-156.011749706061	-156.548655418687
2 × Butadiene	-309.729540133582	-310.751636817418
2 × Hexatriene	-463.448567567639	-464.957123911347
Ethylene + Butadiene	-232.870644919811	-233.650146117761
Ethylene + Hexatriene	-309.730158636977	-310.752889664433
N	-54.332925024884	-54.418481221148
N_2	-108.665850049768	-108.836962442303
F	-99.280473082953	-99.435523849122
F_2	-198.560946165899	-198.871047698237
F_3	-297.841419248868	-298.306571548223

5 Trans-Stilbene

5.1 Energies

Table S2: Trans-Stilbene ground state energies in E_h

RHF	RHF-CCSD	UHF	UHF-CCSD	CASSCF	RIC-MRCCSD
-537.30211	-539.47498	-537.30212	-539.47506	-537.47664	-539.36467

5.2 Structure

```
26
Coordinates from ORCA-job casscf
  C          -5.06766788120857     -1.82485754050615     -9.04498783128743
  C          -4.82415126732170     -0.58143890476712     -8.48637606658008
  C          -4.60366602224727     -2.97636611096314     -8.39567045242089
  H          -5.17931674670346      0.30216163036396     -8.98316443871395
  H          -4.79099787184716     -3.94202102793384     -8.82646416530271
  C          -4.13446903283871     -0.45894739973473     -7.30500259616719
  C          -3.91830605390386     -2.87884738882559     -7.22453464872402
  H          -3.96409969166694      0.51726812098877     -6.90172199082744
  H          -3.56609265410321     -3.76666492284451     -6.73324048196436
  C          -3.64988616611524     -1.61370760099732     -6.62120176891396
  H          -5.60772412422517     -1.90705553799028     -9.96844220299400
  C          -2.94400971762503     -1.55586871079588     -5.42000386961136
  C          -2.61520853623894     -0.36259954017171     -4.72275283193871
  C          -1.90933146347009     -0.30476031269116     -3.52155361190949
  C          -1.42445724670844     -1.45949896241920     -2.83792461281190
  C          -1.64121211903733      0.96035782188975     -2.91804448620406
```

```
H    -1.59452034166247   -2.43569349946267   -3.24138567032198
H    -1.99366053143837    1.84815908148563   -3.40919927164342
C    -0.73480727451557   -1.33700935632884   -1.65653265327268
C    -0.95587942096690    1.05787567354272   -1.74689122663099
H    -0.37941207529260   -2.22059364253871   -1.15987960002389
H    -0.76879435750313    2.02351423114683   -1.31595352911299
C    -0.49160449035612   -0.09361200370102   -1.09773530246345
H     0.04841732570339   -0.01141666111325   -0.17426056121184
H    -2.92632454833431    0.57395954069113   -5.13840155509386
H    -2.63309783337267   -2.49244165632371   -5.00423366385349
```

6 Polyenes

6.1 Energies

Table S3: Ground state energies of polyene series in E_h

Polyene	NEVPT2	CEPA(0)	RIC-MRCCSD
Ethylene	-78.25695	-78.29227	-78.27437
Butadiene	-155.35054	-155.40982	-155.37558
Hexatriene	-232.44588	-232.52886	-232.47816
Octatetraene	-309.54165	-309.64823	-309.58105
Decapentaene	-386.63695	-386.76724	-386.68338
Dodecahexaene	-463.73294	-463.88690	-463.78645
Tetradecaheptaene	-540.81695	-	-540.87692

6.2 Structures

6.2.1 Ethylene

```
6

ethylene from PubChem
```

```
C -0.667200  0.000000  0.000000
C  0.667200  0.000000  0.000000
H -1.221300 -0.929000  0.070800
H -1.221200  0.929000 -0.070800
H  1.221300  0.929000 -0.070800
H  1.221300 -0.929000  0.070800
```

6.2.2 Butadiene

```
10
butadiene from PubChem
C -0.602200  0.397200  0.000000
C  0.602400 -0.397500  0.000000
C -1.831500 -0.130500  0.000000
C  1.831400  0.130800  0.000000
H -0.497500  1.478900  0.000100
H  0.497900 -1.479200  0.000100
H -2.703500  0.515100  0.000000
H -1.997500 -1.202700  0.000000
H  2.703600 -0.514300  0.000000
H  1.996900  1.203000  0.000000
```

6.2.3 Hexatriene

```
14
hexatriene from PubChem
C -0.596800  0.306200  0.000100
C  0.596800 -0.306300  0.000000
C -1.850300 -0.412700  0.000000
```

```
C  1.850200  0.412700 -0.000100
C -3.044100  0.190800  0.000000
C  3.044200 -0.190800  0.000000
H -0.650400  1.392700  0.000000
H  0.650500 -1.392800  0.000000
H -1.813900 -1.498800 -0.000100
H  1.813800  1.498800 -0.000200
H -3.954700 -0.399000 -0.000200
H -3.143100  1.271200  0.000100
H  3.954700  0.399100 -0.000100
H  3.143100 -1.271200  0.000200
```

6.2.4 Octatetraene

```
18
trans-octatetraene from PubChem
C -0.634800  0.346900  0.000900
C  0.634700 -0.347500  0.000700
C -1.816000 -0.289000  0.000900
C  1.816000  0.288800  0.000700
C -3.083300  0.405300 -0.000800
C  3.083400 -0.405100 -0.000800
C -4.265300 -0.221200 -0.000800
C  4.265200  0.221900 -0.000800
H -0.603200  1.434300  0.000300
H  0.603400 -1.434800  0.000100
H -1.848400 -1.376400  0.001300
H  1.848100  1.376200  0.001300
```

H -3.068000 1.491900 -0.002100

H 3.068500 -1.491700 -0.001900

H -5.187100 0.350900 -0.002100

H -4.343200 -1.303400 0.000400

H 5.187300 -0.349800 -0.002000

H 4.342700 1.304100 0.000300

6.2.5 Decapentaene

22

decapentaene from PubChem

C 0.570300 -0.364900 0.001000

C -0.598400 0.295200 0.001000

C 1.850900 0.310900 0.000300

C -1.882100 -0.372800 0.000200

C 3.071200 -0.249400 0.000100

C -3.049700 0.288100 0.000300

C 4.350000 0.425600 -0.000700

C -4.331500 -0.379300 -0.000700

C 5.519200 -0.225600 -0.000700

C -5.500000 0.272100 -0.000700

H 0.589800 -1.452000 0.000700

H -0.609000 1.383000 0.001100

H 1.799800 1.399800 -0.000200

H -1.873100 -1.460600 -0.000600

H 3.123100 -1.338300 0.000600

H -3.059200 1.375800 0.001100

H 4.347000 1.511800 -0.001200

```
H -4.339100 -1.465900 -0.001600
H  6.452100  0.328300 -0.001300
H  5.576500 -1.309000 -0.000100
H -6.433600 -0.280400 -0.001500
H -5.555000  1.355700  0.000200
```

6.2.6 Dodecahexaene

```
26
trans-dodecahexaene from PubChem
C  0.664800  0.341100  0.029400
C -0.640800 -0.283700  0.031800
C  1.834900 -0.316100  0.032700
C -1.811700  0.373600  0.028600
C  3.115500  0.356500 -0.001500
C -3.089700 -0.306600  0.002800
C  4.286000 -0.299600  0.001800
C -4.311900  0.249700 -0.002600
C  5.564800  0.372200 -0.033000
C -5.587900 -0.429500 -0.028300
C  6.735700 -0.275000 -0.030000
C -6.759600  0.217200 -0.031700
H  0.666900  1.429900  0.012100
H -0.641600 -1.372600  0.025700
H  1.844000 -1.403500  0.048500
H -1.830800  1.460400  0.033300
H  3.102800  1.443900 -0.030400
H -3.035200 -1.395200 -0.013200
```

```
H  4.299800 -1.386900  0.031100
H -4.367600  1.338300  0.013800
H  5.568300  1.458500 -0.062900
H -5.580900 -1.515500 -0.045400
H  7.667200  0.280600 -0.057100
H  6.794800 -1.357900 -0.000900
H -7.690300 -0.340000 -0.051400
H -6.821100  1.300300 -0.015300
```

6.2.7 Tetradecaheptaene

```
30
tetradecaheptaene from PubChem
C  0.577800 -0.308800  0.001300
C -0.631400  0.274300  0.001000
C  1.877800  0.325000  0.005300
C -1.918800 -0.387600 -0.002900
C  3.085600 -0.261800  0.005500
C -3.081300  0.283000 -0.003000
C  4.388800  0.366900  0.009400
C -4.370300 -0.374300 -0.002000
C  5.560100 -0.287600  0.009500
C -5.532500  0.296100 -0.002000
C  6.836800  0.388600 -0.010700
C -6.819700 -0.360300 -0.000300
C  8.009700 -0.254700 -0.010800
C -7.982400  0.301300 -0.000200
H  0.611300 -1.398400 -0.001700
```

```
H -0.666200  1.363500  0.003900
H  1.847200  1.414400  0.008200
H -1.910800 -1.474900 -0.003900
H  3.110000 -1.351900  0.002900
H -3.082200  1.370800 -0.001700
H  4.383700  1.455400  0.002600
H -4.370500 -1.462200 -0.001000
H  5.573300 -1.375100  0.014900
H -5.532800  1.383900 -0.003000
H  6.837300  1.475200 -0.025700
H -6.836700 -1.446900  0.000900
H  8.939500  0.304100 -0.026100
H  8.072200 -1.337800  0.004000
H -8.920900 -0.243100  0.001100
H -8.028000  1.385200 -0.001400
```

7 Ethylene Rotation

7.1 ORCA Input

The following ORCA input file can be used to reproduce the ethylene PES scans using the desired method, illustrated here for the FIC-MRCC method. Once the RIC-MRCCSD method is available in the next major release of ORCA, this script can be used to reproduce those results.

```
! def2-TZVP LargePrint NoPop fic-mrcc
%casscf
  nel         2
  norb        2
```

```
  mult        1
  nroots      1
end
%method
  scanguess pmodel
end
%paras
  cc2 = 1.339
  hc3 = 1.087
  dih4 = 180.000
  hcc5 = 121.300
  dih5 = 180.000
  dih6 = 0.000 rot = 0,180,19
end
*int 0 1
  c 0 0 0 0 0 0
  c 1 0 0 {cc2} 0 0
  h 1 2 0 {hc3} {hcc5} 0
  h 1 2 3 {hc3} {hcc5} {dih4}
  h 2 1 3 {hc3} {hcc5} {dih5+rot}
  h 2 1 3 {hc3} {hcc5} {dih6+rot}
*
```

Table S4: Energies in kcal/mol for the ethylene rotation. Since the PES is symmetric, only the first half is reported.

Angle	0.0	10.0	20.0	30.0	40.0	50.0	60.0	70.0	80.0	90.0
CASSCF	-49003.4	-49002.1	-48998.3	-48992.0	-48983.4	-48972.6	-48960.0	-48946.6	-48934.5	-48929.0
CEPA0	-49238.1	-49236.9	-49233.3	-49227.3	-49219.1	-49208.8	-49196.8	-49183.7	-49171.6	-49165.9
MRCC	-49232.1	-49230.8	-49227.2	-49221.2	-49212.9	-49202.5	-49190.2	-49176.9	-49164.5	-49158.6
NEVPT2	-49213.8	-49212.6	-49208.9	-49202.7	-49194.2	-49183.5	-49170.9	-49157.0	-49144.0	-49137.8
NEVPT3	-49229.8	-49228.6	-49224.9	-49218.8	-49210.4	-49199.9	-49187.6	-49174.3	-49162.1	-49156.5
NEVPT4	-49235.9	-49234.7	-49231.1	-49225.1	-49216.7	-49206.3	-49194.0	-49180.7	-49168.4	-49162.6
RIC-MRCCSD ($s = 0.5$)	-49223.2	-49221.9	-49218.1	-49211.9	-49203.3	-49192.4	-49179.5	-49165.2	-49151.6	-49144.9
RIC-MRCCSD ($s = 2.2$)	-49232.7	-49231.4	-49227.8	-49221.7	-49213.2	-49202.6	-49190.1	-49176.7	-49164.7	-49159.3
RIC-MRCCSD ($s = 8.0$)	-49232.1	-49230.8	-49227.1	-49221.0	-49212.7	-49202.3	-49190.3	-49177.8	-49167.2	-49162.9

7.2 Energies

8 Vitamin B12 Model

8.1 Energies

Table S5: Ground state energy in E_h of Vitamin B12 model.

CASSCF	NEVPT2	NEVPT4	RHF-CCSD	RIC-MRCCSD
-2889.12900	-2894.79478	-2895.13962	-2894.97960	-2894.72500

8.2 Structure

The molecular structure of the Vitamin B12 model studied in the manuscript is taken from the supporting information of Ref. 6, which is derived from high-resolution X-ray crystallographic data. It is reported here for convenience.

```
68

Co    -0.97688200000000     0.01491400000000     0.39430300000000
N     -0.13142600000000     1.59419600000000     0.98032000000000
N      0.03482200000000    -1.04279600000000     1.66105700000000
N     -1.92361200000000    -1.49721200000000    -0.36583900000000
```

N	-1.98261500000000	1.22050600000000	-0.63675500000000
C	-0.41224400000000	2.82915200000000	0.20025900000000
C	-0.08743500000000	3.97388700000000	1.18675700000000
C	0.89972700000000	3.31916600000000	2.18892300000000
C	0.56842000000000	1.84246200000000	2.06668100000000
C	0.98045100000000	0.81501900000000	2.96579400000000
C	0.72341200000000	-0.52282700000000	2.75164900000000
C	1.15017900000000	-1.63817200000000	3.68767200000000
C	0.87400000000000	-2.91384900000000	2.86566100000000
C	0.00518900000000	-2.39986900000000	1.73461300000000
C	-0.74824300000000	-3.23017100000000	0.90100100000000
C	-1.67400200000000	-2.79802400000000	-0.05031500000000
C	-2.53876700000000	-3.74671100000000	-0.85829100000000
C	-3.49821100000000	-2.79939800000000	-1.60773600000000
C	-2.94368200000000	-1.41819900000000	-1.30464900000000
C	-3.40308400000000	-0.25559700000000	-1.89491600000000
C	-2.93440800000000	1.04019800000000	-1.52930100000000
C	-3.46848800000000	2.37424700000000	-2.02970400000000
C	-2.40110900000000	3.37826200000000	-1.51335600000000
C	-1.85871800000000	2.65446800000000	-0.26812600000000
N	1.16919000000000	-0.48413500000000	-3.21765400000000
C	0.09084900000000	-0.38583200000000	-2.38635700000000
N	0.44699000000000	-0.19623600000000	-1.11431200000000
C	2.79593700000000	0.01261200000000	-0.08354500000000
C	4.16439000000000	-0.00050900000000	-0.39124400000000
C	4.61294200000000	-0.19117500000000	-1.74229300000000
C	3.68095500000000	-0.36488300000000	-2.77503200000000

C	2.31901700000000	-0.34762300000000	-2.44427000000000
C	1.85430800000000	-0.16388000000000	-1.11541100000000
C	5.17909600000000	0.18863500000000	0.71675200000000
C	6.09214200000000	-0.20650700000000	-2.05974600000000
H	0.78242600000000	3.68355800000000	3.22368400000000
H	0.37139500000000	-3.71089200000000	3.43795900000000
H	-0.66279300000000	-4.30908600000000	1.06021800000000
H	-4.53101100000000	-2.87217000000000	-1.22031500000000
H	-1.59676800000000	3.50115200000000	-2.26246600000000
H	-2.52426100000000	2.82238100000000	0.60487700000000
H	-0.93538800000000	-0.45576700000000	-2.74405000000000
H	2.47429800000000	0.15995900000000	0.95045100000000
H	4.01614000000000	-0.50902300000000	-3.80796900000000
H	4.68387100000000	0.31977600000000	1.69324300000000
H	5.81677800000000	1.07539400000000	0.54150000000000
H	5.86220100000000	-0.67755800000000	0.79875000000000
H	6.58242600000000	0.74110100000000	-1.76794500000000
H	6.26934500000000	-0.35857600000000	-3.13705600000000
H	6.61726400000000	-1.01371600000000	-1.51540200000000
H	1.12910900000000	-0.63023600000000	-4.22494500000000
H	-1.00766300000000	4.28229600000000	1.71487500000000
H	1.95666700000000	3.48742300000000	1.90310000000000
H	1.52565000000000	1.10219400000000	3.86882000000000
H	2.19885600000000	-1.53150100000000	4.01036500000000
H	0.52271000000000	-1.60689100000000	4.59827900000000
H	1.80498200000000	-3.34706200000000	2.45193800000000
H	-3.05890700000000	-4.46721200000000	-0.20432300000000

H	-1.90734600000000	-4.33873600000000	-1.54716700000000
H	-3.54931800000000	-2.99105100000000	-2.69296900000000
H	-4.20460200000000	-0.33100300000000	-2.63486900000000
H	-3.60799500000000	2.39112700000000	-3.12428500000000
H	-4.45988900000000	2.56450800000000	-1.57282400000000
H	-2.81822100000000	4.37197200000000	-1.28666900000000
H	0.33174500000000	4.86039900000000	0.68557500000000
H	0.26127700000000	2.83988500000000	-0.68179400000000
C	-2.27135700000000	0.17669900000000	1.69611900000000
N	-3.10974400000000	0.29355800000000	2.52028400000000

References

(1) Lechner, M. H.; Papadopoulos, A.; Sivalingam, K.; Auer, A. A.; Koslowski, A.; Becker, U.; Wennmohs, F.; Neese, F. Code Generation in ORCA: Progress, Efficiency and Tight Integration. *Phys. Chem. Chem. Phys.* **2024**, *26*, 15205–15220.

(2) Hanauer, M.; Köhn, A. Pilot Applications of Internally Contracted Multireference Coupled Cluster Theory, and How to Choose the Cluster Operator Properly. *J. Chem. Phys.* **2011**, *134*, 204111.

(3) Sivalingam, K.; Krupicka, M.; Auer, A. A.; Neese, F. Comparison of fully internally and strongly contracted multireference configuration interaction procedures. *J. of Chem. Phys.* **2016**, *145*, 054104.

(4) Malmqvist, Per-Åke Calculation of transition density matrices by nonunitary orbital transformations. *Int. J. Quantum Chem.* **1986**, *30*, 479–494.

(5) Scuseria, G. E.; Lee, T. J.; Schaefer III, H. F. Accelerating the convergence of the coupled-cluster approach: The use of the DIIS method. *Chemical Physics Letters* **1986**, *130*, 236–239.

(6) Kornobis, K.; Kumar, N.; Wong, B. M.; Lodowski, P.; Jaworska, M.; Andruniów, T.; Ruud, K.; Kozlowski, P. M. Electronically Excited States of Vitamin B12: Benchmark Calculations Including Time-Dependent Density Functional Theory and Correlated ab Initio Methods. *J. Phys. Chem. A* **2011**, *115*, 1280–1292.
